# Spontaneous generation of singlet oxygen on microemulsion-derived manganese oxides with rich oxygen vacancies for efficient aerobic oxidation†

**DOI:** 10.1039/d3sc04418a

**Published:** 2023-10-12

**Authors:** Jun Tang, Junbao Chen, Zhanyu Zhang, Qincheng Ma, Xiaolong Hu, Peng Li, Zhiqiang Liu, Peixin Cui, Chao Wan, Qingping Ke, Lei Fu, Jeonghun Kim, Takashi Hamada, Yunqing Kang, Yusuke Yamauchi

**Affiliations:** a College of Chemistry and Chemical Engineering, Anhui University of Technology Maanshan 243002 Anhui P. R. China Qingke@ahut.edu.cn; b School of Chemistry and Chemical Engineering, Shanxi University Taiyuan 030006 P. R. China; c Key Laboratory of Soil Environment and Pollution Remediation, Institute of Soil Science, The Chinese Academy of Sciences Nanjing 210008 P. R. China; d College of Chemical and Biological Engineering, Zhejiang University Hangzhou 310058 P. R. China wanchao@zju.edu.cn; e Research Center for Materials Nanoarchitectonics (MANA), National Institute for Materials Science (NIMS) 1-1 Namiki Tsukuba Ibaraki 305-0044 Japan yqkang@toki.waseda.jp; f Department of Chemical and Biomolecular Engineering, Yonsei University 50 Yonsei-ro, Seodaemun-gu Seoul 03722 South Korea; g Department of Materials Process Engineering, Graduate School of Engineering, Nagoya University Nagoya 464-8603 Japan; h Australian Institute for Bioengineering and Nanotechnology (AIBN), The University of Queensland Brisbane Queensland 4072 Australia y.yamauchi@uq.edu.au

## Abstract

Developing innovative catalysts for efficiently activating O_2_ into singlet oxygen (^1^O_2_) is a cutting-edge field with the potential to revolutionize green chemical synthesis. Despite its potential, practical implementation remains a significant challenge. In this study, we design a series of nitrogen (N)-doped manganese oxides (N_*y*_-MnO_2_, where *y* represents the molar amount of the N precursor used) nanocatalysts using compartmentalized-microemulsion crystallization followed by post-calcination. These nanocatalysts demonstrate the remarkable ability to directly produce ^1^O_2_ at room temperature without the external fields. By strategically incorporating defect engineering and interstitial N, the concentration of surface oxygen atoms (O_s_) in the vicinity of oxygen vacancy (O_v_) reaches 51.1% for the N_55_-MnO_2_ nanocatalyst. This feature allows the nanocatalyst to expose a substantial number of O_v_ and interstitial N sites on the surface of N_55_-MnO_2_, facilitating effective chemisorption and activation of O_2_. Verified through electron paramagnetic resonance spectroscopy and reactive oxygen species trapping experiments, the spontaneous generation of ^1^O_2_, even in the absence of light, underscores its crucial role in aerobic oxidation. Density functional theory calculations reveal that an increased O_v_ content and N doping significantly reduce the adsorption energy, thereby promoting chemisorption and excitation of O_2_. Consequently, the optimized N_55_-MnO_2_ nanocatalyst enables room-temperature aerobic oxidation of alcohols with a yield surpassing 99%, representing a 6.7-fold activity enhancement compared to ε-MnO_2_ without N-doping. Furthermore, N_55_-MnO_2_ demonstrates exceptional recyclability for the aerobic oxidative conversion of benzyl alcohol over ten cycles. This study introduces an approach to spontaneously activate O_2_ for the green synthesis of fine chemicals.

## Abstract 2

We fabricated a range of N_*y*_-MnO_*x*_ using compartmentalized microemulsion crystallization followed by post-calcination. These materials have the unique ability to generate sufficient ^1^O_2_ at room temperature without requiring an external field.

## Graphical abstract

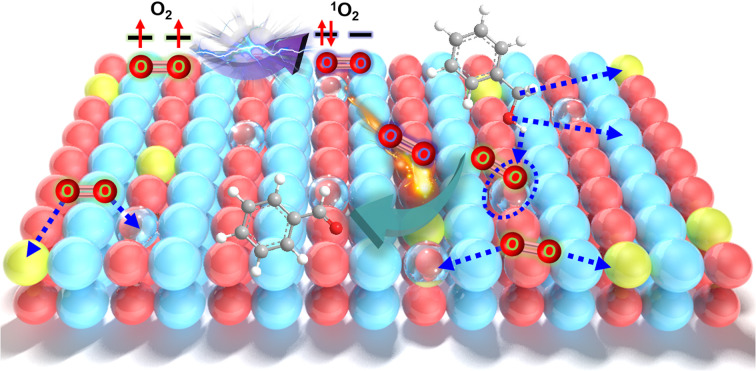

## Introduction

Singlet oxygen (^1^O_2_), recognized as a highly active and environmentally friendly reactive oxygen species (ROS), has garnered substantial attention from researchers in recent years.^1–4^ Benefiting from its remarkable oxidative capabilities and eco-friendliness, ^1^O_2_ has found widespread applications in green catalysis,^5–7^ photocatalytic degradation,^8^ tumor diagnosis and treatment,^9^ and fluorescence probes.^10^ Nevertheless, the practical generation of ^1^O_2_ typically necessitates intense photoexcitation due to the spin transition between ground and excited state molecular oxygen.^11^ Throughout the last few decades, a range of photosensitizers, including photosensitive organic dyes,^12^ organometallic complexes,^13^ and noble metals,^14,15^ have been devised to produce ^1^O_2_ by harnessing photogenerated excitons and energy transfer pathways. However, the scarcity of noble metals and the susceptibility of organic photosensitizers to degradation render them inadequate to fulfill the escalating needs of green chemistry and sustainable development.^11,16^ Therefore, the construction of cost-effective and stable triggers for self-activating O_2_ in a mild environment through catalyst innovation holds great significance.

Transition metal oxides are being considered as suitable candidates to replace noble metal catalysts due to their significant advantages, including low cost, excellent stability, and abundant reserves.^17–20^ Among these, manganese dioxides (MnO_2_) stand out due to the multiple valence states and intricate electronic structures, which can be readily modified through structural-tailoring strategies to enhance their redox capabilities. Our previous studies have demonstrated the effectiveness of *in situ* heteroatom insertion techniques in producing highly active catalysts with ample oxygen vacancies (O_v_).^17,19^ These O_v_ play a pivotal role in promoting the mobility of lattice oxygen (O_l_), consequently enhancing oxidative capability through the Mars–Van Krevelen (MVK) mechanism, rather than inducing spontaneous O_2_ transformation into ^1^O_2_.^21^ While theoretical studies have suggested that O_2_ adsorbed on the surface of metal oxides can lead to the direct generation of ^1^O_2_, experimental breakthroughs in this area have been limited.^22–24^ Fortunately, the defect-engineering approach, particularly by modulating rich O_v_, promotes the chemisorption of O_2_, offering a promising avenue for activating O_2_ to form ROS.^25–31^ For instance, the {111} facet of MgO, with its abundance of O_v_, enables chemisorbed O_2_ to undergo molecule transitions and electron rearrangements, resulting in the production of ^1^O_2_ even in the absence of light. However, the application in catalysis remains rarely explored.^24^ It can be inferred that an increase in O_v_ content facilitates O_2_ chemisorption on the surface of the catalyst, thereby promoting the formation of ^1^O_2_.^24,25^ These inspire us to devise catalysts with enhanced activity by creating ample surface chemisorption sites through innovative defect-engineering strategies.

The microemulsion composed of immiscible oleic and aqueous phases serves as a versatile platform for fabricating various nanocatalysts, distinguishing it significantly from conventional methods.^32,33^ Additionally, the utilization of microemulsions as templates for MnO_2_ preparation offers unique advantages: (1) confined aqueous microdroplets create a conducive environment for effective heteroatom doping and the formation of abundant O_v_; (2) uniformly sized MnO_2_ nanocatalysts formed within these microdroplets tend to expose a higher proportion of surface O_v_.^32,33^ However, the strategy of constructing high-concentration O_v_ based on a microemulsion microreactor is rarely reported.^25^

In this study, we synthesized N-doped MnO_2_ (N_*y*_-MnO_2_, where *y* represents the molar amount of urea used) nanocatalysts with adjustable O_v_ through crystallization within compartmentalized droplets of a microemulsion, followed by calcination. The resulting N_*y*_-MnO_2_ nanocatalysts are employed to activate O_2_ and generate ^1^O_2_ for green oxidation processes. As demonstrated in the aerobic oxidation of benzyl alcohol, an important reaction pathway for the synthesis of fine chemicals, the catalytic activity of N_55_-MnO_2_ at room temperature significantly outperforms that of pristine ε-MnO_2_ and commercially activated MnO_2_ (C-MnO_2_). We further elucidate how O_v_ promote O_2_ chemisorption and spontaneous activation through structural characterizations and density functional theory (DFT) calculations. Moreover, the pivotal roles of ROS in the oxidation process through controlled quenching experiments are also verified. This work offers a practical strategy and theoretical insights for constructing environmentally friendly Mn-based catalysts and producing highly desirable ^1^O_2_ for efficient aerobic oxidation.

## Results and discussion

### Synthesis and characterization

**Scheme 1:**
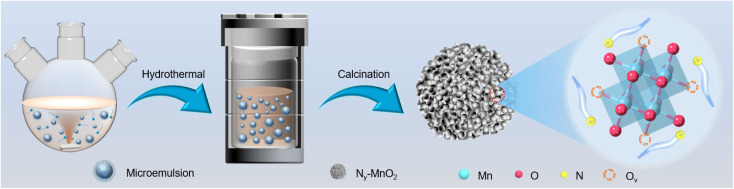
Schematic illustration of the preparation of N_*y*_-MnO_2_ nanocatalysts using compartmentalized microdroplets.

**Fig. 1:**
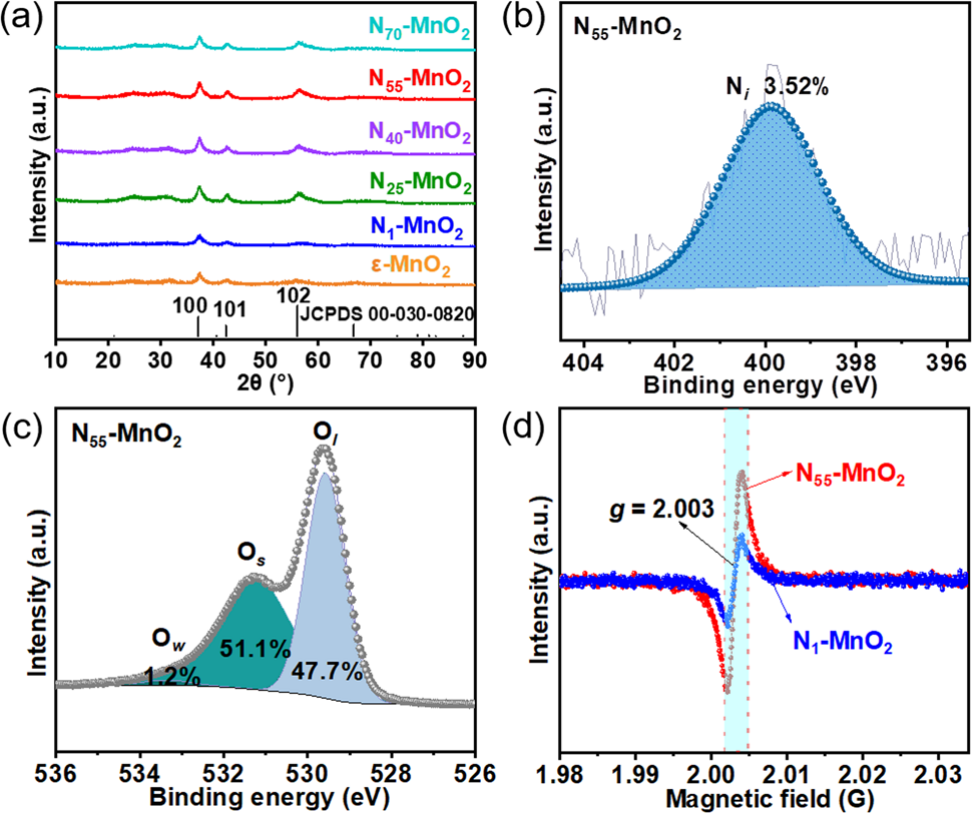
(a) XRD patterns of N_*y*_-MnO_2_ (*y* = 1, 25, 40, 55, 70) and ε-MnO_2_; (b) N 1s and (c) O 1s XPS spectra of N_55_-MnO_2_ (O_w_: oxygen from water adsorption); (d) EPR spectra of N_1_-MnO_2_ and N_55_-MnO_2_.

**Fig. 2:**
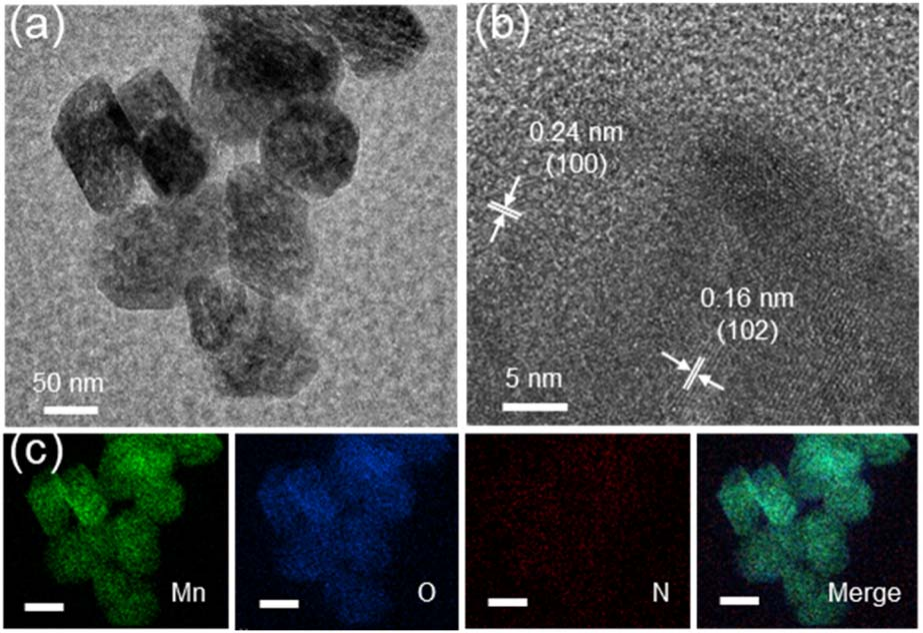
(a)TEM, (b) HRTEM, and (c) EDS elemental mapping images (scale bars, 50 nm) of N_55_-MnO_2_ nanocatalyst.

Compartmentalized microemulsions, stabilized by an excess of surfactants, serve as versatile miniature reactors for the synthesis of N_*y*_-MnO_2_ to spontaneously produce ^1^O_2_ (Scheme 1). Within the confined aqueous microdroplets, isonitrile acid produced by urea decomposition reacts with Mn^2+^ to form catalyst precursor, manganese carbonate (MnCO_3_), as verified by X-ray diffraction (XRD) patterns in Fig. S1a.† Fourier transform infrared (FTIR) spectra in Fig. S1b† display the presence of new peaks at 1443 cm^−1^, 866 cm^−1^, and 720 cm^−1^, further confirming the successful formation of MnCO_3_.^33,34^ Following calcination in air, MnCO_3_ decomposes into MnO_2_. The XRD patterns of the N_*y*_-MnO_2_ nanocatalysts, as shown in Fig. 1a, are identified as ε-MnO_2_. The disappearance of the broad band at 1443 cm^−1^ and the emergence of peak at 1385 cm^−1^ in FTIR spectra indicates the incorporation of N atoms into ε-MnO_2_ (Fig. S1b†). Additionally, X-ray photoelectron spectroscopy (XPS) was employed to analyze the embedded N in N_55_-MnO_2_. The distinct peak at 399.6 eV in the N 1s spectra of N_*y*_-MnO_2_ (Fig. 1b and S2†) is unequivocally attributed to N atoms inserted at interstitial sites.^35^ The introduction of interstitial N minimizes the formation energy of O_v_ and facilitates the generation of high-concentration O_v_.^35^ As a result, the N_55_-MnO_2_ catalyst, with an optimal interstitial N content, achieves a maximum concentration of surface oxygen atoms (O_s_) at 51.1% in the vicinity of O_v_ (Fig. 1c, S3, and Table S1†).^36,37^ In addition, the O_s_ content and the unsaturated Mn coordination (Mn^3+^/Mn^4+^ ratio of 0.90, as shown in Fig. S4†), in N_55_-MnO_2_, significantly exceed that of similar catalysts (Table S2†).^37–41^ Furthermore, the evolutionary trend facilitated by interstitial N-doping is reflected in the increased-intensity peak of O_v_ at *g* = 2.003 in the electron paramagnetic resonance (EPR) spectrum in Fig. 1d. However, excessive N-doping in N_70_-MnO_2_ tends to occupy the formed vacancies, negatively affecting O_v_ formation (Fig. S3e†). As observed by transmission electron microscopy (TEM) in Fig. 2a, the N_55_-MnO_2_ catalyst possesses a size of ∼115 nm, which is consistent with the results from dynamic light scattering (DLS, Fig. S1c†). This demonstrates the distinctive compartmentalization effect of microemulsions in synthesizing uniform-size nanocatalysts. Additionally, High-resolution TEM (HRTEM) image of N_55_-MnO_2_ reveals *d*-spacings of 0.24 nm and 0.16 nm (Fig. 2b), corresponding to the (100) and (102) facets, respectively, of ε-MnO_2_.^42^ Energy dispersive spectroscopy (EDS) element mapping images of N_55_-MnO_2_ demonstrate that interstitial N is uniformly embedded in the MnO_2_ lattice (Fig. 2c). Doping interstitial N into MnO_2_ leads to alterations in the original electronic structure and unsaturated coordination environment, thus enhancing the catalytic oxidation capacity.^37^ This is confirmed through hydrogen temperature-programmed reduction (H_2_-TPR), with N_55_-MnO_2_ displaying the most prominent redox capability (Fig. S1d†), consistent with previous reports.^37,43–47^ Additionally, Brunauer–Emmett–Teller (BET) analysis of N_2_ adsorption–desorption measurements was conducted to assess the BET parameters of N_*y*_-MnO_2_. As shown in Fig. S5 and Table S3,† N_55_-MnO_2_ exhibits the highest BET surface area of 78.7 m^2^ g^−1^ among the N_*y*_-MnO_2_ samples and an average mesopore size of 11.41 nm. This can be attributed to the presence of O_v_ in N_*y*_-MnO_2_, which contribute to the formation of abundant mesopores on the surface of N_*y*_-MnO_2_.^48^

### Adsorption of O_2_ and production of ^1^O_2_

**Fig. 3:**
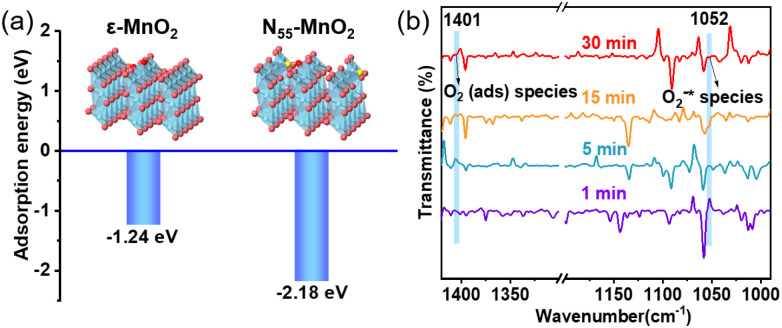
(a) Adsorption energy of O_2_ on the surface of pristine ε-MnO_2_, and N_55_-MnO_2_ obtained by DFT calculation; (b) *in situ* IR spectra of O_2_ adsorbed onto the surface of N_55_-MnO_2_.

Previous studies have demonstrated that an increased concentration of O_v_ enhances the chemical adsorption of O_2_, leading to the elongation of the O–O bond and facilitating O_2_ activation.^24,25,48^ Our DFT calculations reveal that the adsorption energy of O_2_ on the surface of ε-MnO_2_ is −1.24 eV, whereas it decreases to −2.18 eV on N_55_-MnO_2_ (Fig. 3a). This indicates that the augmented O_v_ resulting from N-doping reduces the adsorption energy of O_2_, thereby promoting its chemisorption and excitation.^25^ To gain further insights, we employed *in situ* IR spectroscopy for real-time monitoring of O_2_ adsorption and activation on the surface of N_55_-MnO_2_. As shown in Fig. 3b, a chemisorption peak of O_2_ emerges at 1401 cm^−1^ in an O_2_ atmosphere, followed by the gradual formation of a robust band corresponding to surface-bonded superoxide anion species (O_2_^−^*) at 1052 cm^−1^.^49,50^ This illustrates the role of O_v_ in promoting O_2_ activation. Conversely, no discernible peaks are observed under an O_2_ + H_2_O vapor atmosphere, possibly due to H_2_O occupying the O_v_ and preventing O_2_ adsorption (Fig. S6a†).

**Fig. 4:**
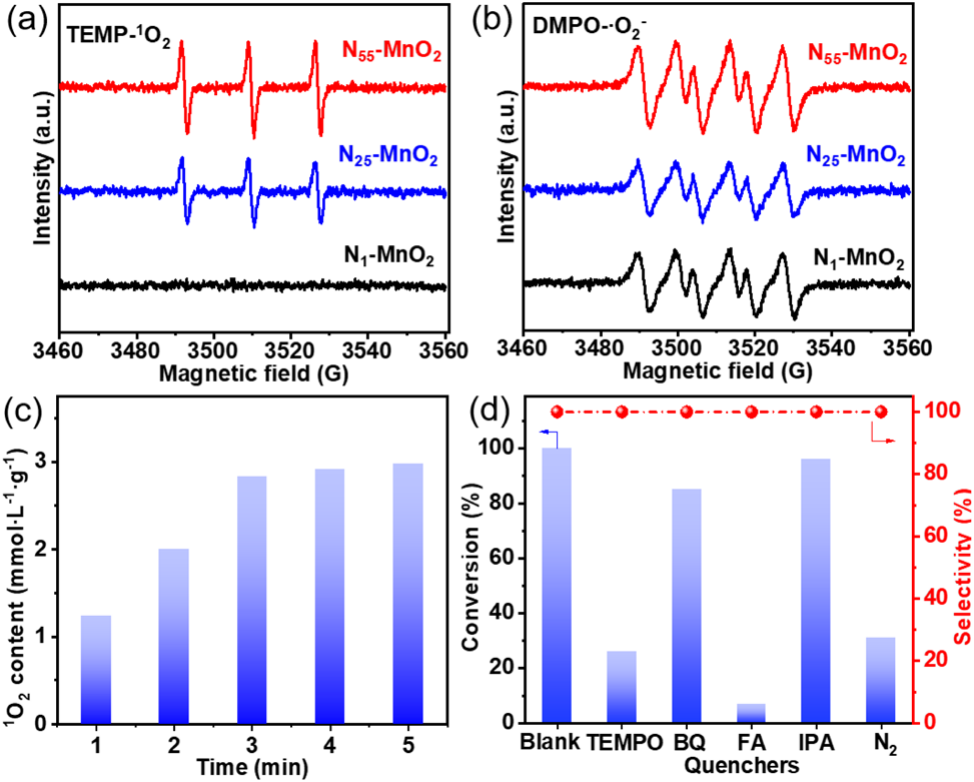
The characteristic peaks of (a) TEMP-^1^O_2_ and (b) DMPO-˙O_2_^−^ captured by EPR; (c) the content of ^1^O_2_ produced by N_55_-MnO_2_; (d) the controlling experiment catalyzed by N_55_-MnO_2_ with different quenchers.

The direct interactions between O_2_ and N_*y*_-MnO_2_ were investigated without light irradiation using EPR. 2,2,6,6-tetramethylpiperidine (TEMP) was employed as a quencher to capture ^1^O_2_ activated by N_*y*_-MnO_2_ at room temperature. As depicted in Fig. 4a and b, N_1_-MnO_2_, which exhibits fewer O_v_, generates only ˙O_2_^−^ and not ^1^O_2_. With an increase in urea feeding, enhanced N-doping promotes O_v_ formation in N_*y*_-MnO_2_. Notably, distinct triple peaks corresponding to TEMP-^1^O_2_ are evident for both N_55_-MnO_2_ and N_25_-MnO_2_, with an intensity ratio of 1 : 1 : 1.^25^ The intensity of the ^1^O_2_ peak strengthens with higher O_v_ content, underscoring the significant role of O_v_ in activating O_2_. Quantitative EPR analysis indicates that N_55_-MnO_2_ generates ^1^O_2_ at an average rate of 5.94 mol g_cat_^−1^ L^−1^ min^−1^ (Fig. 4c). Importantly, N_*y*_-MnO_2_ doesn't produce highly reactive hydroxyl radicals (˙OH, Fig. S6†).^51,52^ To investigate the contribution of O_v_ to the excitation of O_2_, various ε-MnO_2_ nanocatalysts were synthesized and characterized without N doping (Fig. S7–S9†). Interestingly, ε-MnO_2_-*n*-350 (where 350 indicates the thermal treatment temperature), with the highest O_s_ concentration of 44.4%, demonstrates the capability to generate ^1^O_2_ under mild conditions (Fig. S10†). These findings underscore the pivotal role played by abundant surface-exposed O_v_ in nano-sized MnO_2_ for the self-activation of O_2_. However, the catalytic activity of ε-MnO_2_-n-350 is still much lower than that of N_55_-MnO_2_ can be attributed to the doping effect of interstitial N, which will be further discussed later.

To investigate the roles of ROS in the reaction, controlled quenching experiments were conducted. As shown in Fig. 4d, the conversion of benzyl alcohol sharply decreases from >99.9% to 26% after the addition of TEMPO as a ROS quencher, indicating that ROS mediate the oxidative reaction. Upon separately introducing 1,4-benzoquinone (BQ) and isopropyl alcohol (IPA), the conversion of benzyl alcohol decreases slightly, illustrating that ˙O_2_^−^ and ˙OH play subsidiary roles in the selective oxidation process. Surprisingly, the oxidation reaction is nearly halted when furfuryl alcohol (FA) is used to capture ^1^O_2_. This undeniable evidence confirms ^1^O_2_ as the primary ROS in this O_2_-mediated oxidative reaction. In summary, ^1^O_2_ is spontaneously generated through the direct activation of O_2_, rather than the oxidation of ˙O_2_^−^ observed in photocatalytic processes.^52^ Despite O_2_ being the source of ROS, the conversion of benzyl alcohol can be achieved at 31% and 25.5% under N_2_ and Ar atmospheres, respectively (Fig. 4d, S11, and Table S4†). This is attributed to the involvement of O_l_ in the aerobic oxidation process, following the MVK mechanism, which is consistent with previous reports.^17,44^

### Selective oxidation of alcohols

**Fig. 5:**
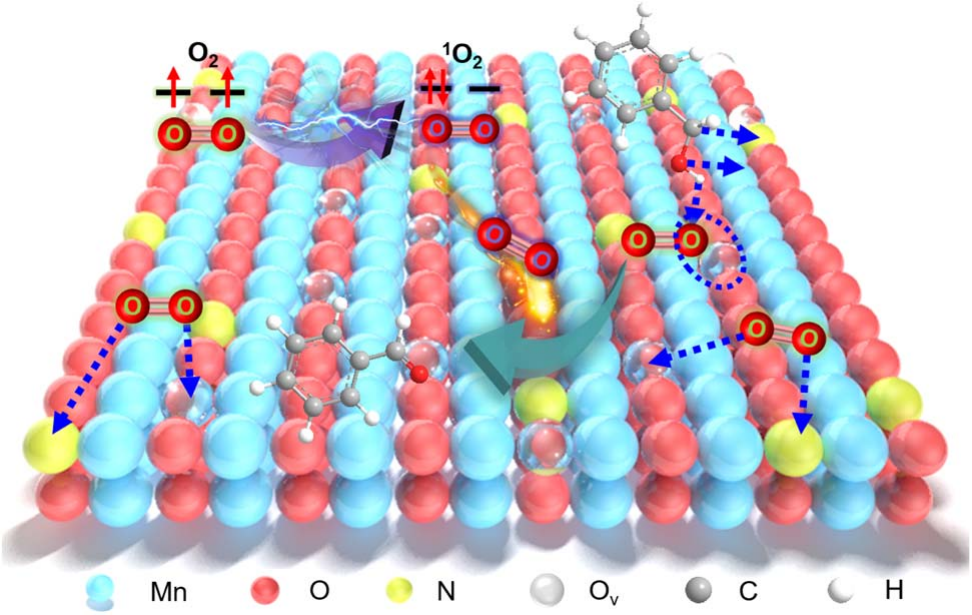
Plausible reaction process for efficient oxidation by N_*y*_-MnO_2_ activating O_2_ to form ^1^O_2_.

**Fig. 6:**
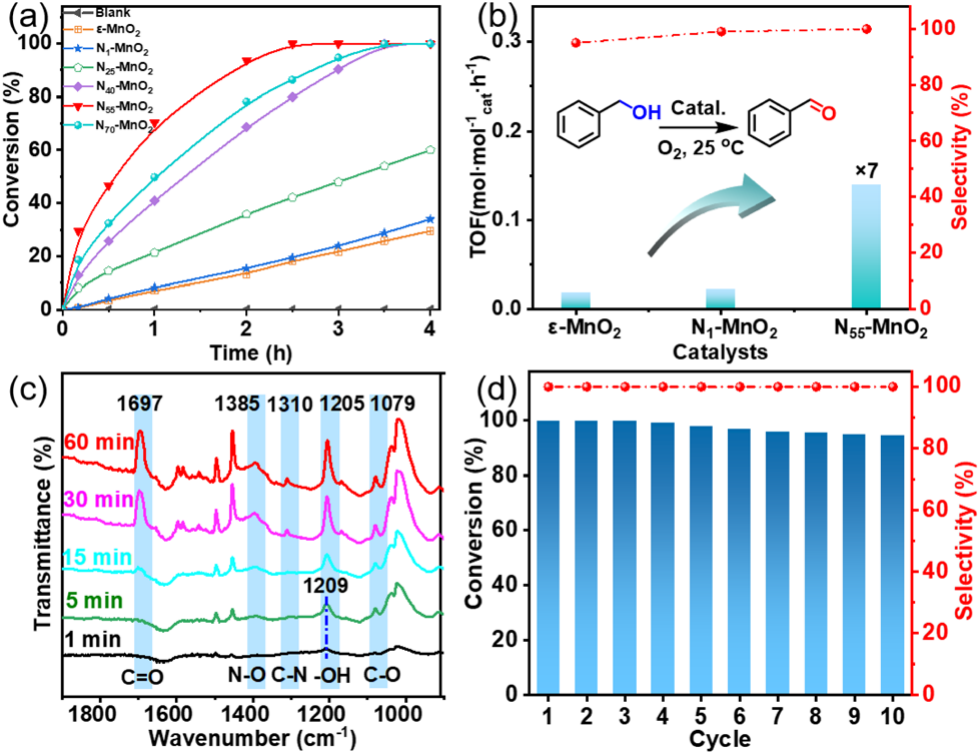
(a) Aerobic oxidation of benzyl alcohol using activated ε-MnO_2_ and N_*y*_-MnO_2_ (*y* = 1, 25, 40, 55, 70), (b) the comparison of TOF of ε-MnO_2_, N_1_-MnO_2_, and N_55_-MnO_2_ (TOF = moles of benzyl alcohol converted per mole of catalyst/reaction time); (c) *in situ* IR spectra of oxidation of benzyl alcohol on the surface of N_55_-MnO_2_; (d) the recyclability of N_55_-MnO_2_ for aerobic oxidation of benzyl alcohol.

Aerobic oxidation of alcohols serves as a widely important model reaction for assessing the oxidative activity of the catalyst. However, effectively exciting O_2_ with catalysts remains a challenge.^15,20^ If a catalyst can spontaneously generate ^1^O_2_ from the ground-state oxygen, it will significantly advance the development of environmentally friendly catalysis. The plausible reaction process for efficient oxidation by N_*y*_-MnO_2_ is presented in Fig. 5. O_v_ and interstitial N sites promote O_2_ adsorption on the surface of N_*y*_-MnO_2_, while O_v_ elongate the O–O bonds, facilitating direct excitation of O_2_ to ^1^O_2_ for efficient alcohol oxidation. We evaluate the catalytic performance of N_*y*_-MnO_2_ for the aerobic oxidation of benzyl alcohol under O_2_ bubbling conditions. We observe that micrometer-scale ε-MnO_2_ displays low activity (conversion rate of 18.2% in 2.5 hours) due to its inability to activate O_2_ to form ^1^O_2_ (Fig. 6a and S10†). The C-MnO_2_ exhibits negligible activity (Fig. S12 and S13†). In contrast, N_55_-MnO_2_ achieves 99.9% conversion and 99.9% selectivity in 2.5 hours, with a turnover frequency (TOF) 7.4 and 6.7 times higher than that of ε-MnO_2_ and ε-MnO_2_-n-350, respectively (Fig. 6b and Table S5†).

To unravel the catalytic reaction mechanism, we conducted kinetics studies using various catalysts. It can be observed that the initial reaction rate remains constant under different O_2_ pressures, indicating independence from O_2_ pressure variations (Fig. S14 and Tables S6–S8†). Through calculation and fitting, the reaction is found to be correlated with the concentration of benzyl alcohol, further demonstrating a first-order reaction (Tables S9 and S10†). Furthermore, we calculate the activation energies (*E*_a_) of the N_1_-MnO_2_, N_25_-MnO_2_, and N_55_-MnO_2_ catalysts using the Arrhenius equation, resulting in values of 55.04 kJ mol^−1^, 45.87 kJ mol^−1^, and 43.17 kJ mol^−1^, respectively (Fig. S15 and Tables S11–S13†). Evidently, the catalytic performance of N_*y*_-MnO_2_ is positively correlated with its O_v_ content, providing further evidence of the role of O_v_ in the oxidation process. *In situ* IR spectra were utilized to analyze the dynamic process of benzyl alcohol oxidation on the surface of N_55_-MnO_2_, revealing enhanced catalytic performance. Under an O_2_ atmosphere, N_55_-MnO_2_ serves as the background for data acquisition. In the first five minutes, four characteristic peaks emerge in Fig. 6c. Compared with C-MnO_2_ (Fig. S16†), the newly appeared N–O peak at 1385 cm^−1^ on N_55_-MnO_2_ is attributed to O_2_ adsorption by interstitial N sites. At 1310 cm^−1^, the band corresponds to the stretching vibration of C–N bonds. The formation of C–N bonds is facilitated by the chemisorption of –OH groups from alcohol onto the interstitial N sites. After exposure to an O_2_ flow for 15 minutes, the O–H band shifts from 1209 cm^−1^ to 1205 cm^−1^, indicating an interaction between the adsorbed O_2_ species and -C-OH groups on benzyl alcohol. Continuing the exposure for 30 minutes, the broad C=O band at 1697 cm^−1^ signifies the formation of benzyl aldehyde catalyzed by N_55_-MnO_2_.

Semiconductor catalysts have recently demonstrated an enhanced capacity for generating ^1^O_2_ when exposed to light, resulting in improved oxidation performance.^24,25^ In contrast, N_55_-MnO_2_ exhibits virtually unchanged activity under both light and dark conditions (Table S4†). This observation suggests that the inherent capability of N_55_-MnO_2_ to spontaneously generate ^1^O_2_ operates independently of external factors, marking a significant difference from previously reported studies.^25,50,53,54^ Compared to recently reported heterogeneous catalysts, the well-designed N_*y*_-MnO_2_ nanocatalysts display a unique ability to spontaneously generate sufficient ^1^O_2_ under mild reaction conditions. This enables the green and efficient conversion of alcohols without the need for light or additives, showcasing their superior catalytic activity (Table S14†).

Furthermore, we investigated the applicable substrate scope of aerobic oxidation. As shown in Table 1, aromatic primary alcohols, including benzyl alcohol and its derivatives, exhibit yields of over 99.9% for the corresponding aldehydes. Even for aromatic secondary alcohols, yields exceeding 99.9% are obtained when the reaction time extends to 6.0 hours. In comparison, the catalytic performance of N_55_-MnO_2_ in the oxidation of aliphatic alcohols (*e.g.*, 1-hexanol) is hampered by significant steric hindrance effects. Using benzyl alcohol as the model substrate, we evaluate the recyclability of N_55_-MnO_2_ in aerobic oxidation. As depicted in Fig. 6d, N_55_-MnO_2_ maintains >94% conversion of benzyl alcohol and >99.9% selectivity toward aldehydes over ten cycles. Additionally, XRD and XPS characterizations confirm the stability of the recycled N_55_-MnO_2_ structure (Fig. S17†). To the best of our knowledge, results demonstrating both sufficient formation of ^1^O_2_ and the green oxidation of alcohols under mild conditions have been scarcely reported.

**Table 1:** Catalytic activity of N_55_-MnO_2_ for aerobic oxidation of various alcohols^a^

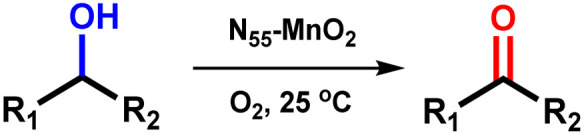				
Entry	Product	Conv. (%)	Sel. (%)	Yield (%)
1	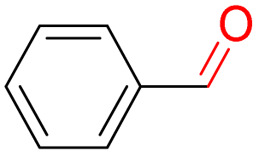	99.9	>99.9	>99.9
2	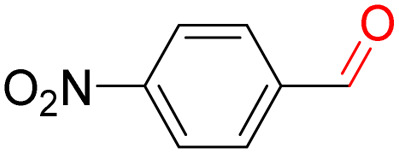	99.9	>99.9	>99.9
3	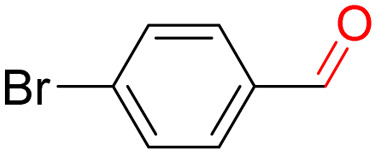	99.9	>99.9	>99.9
4	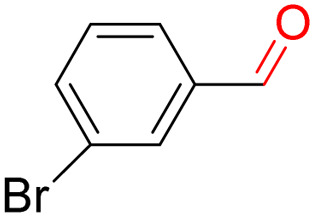	82.6	>99.9	>82.6
5	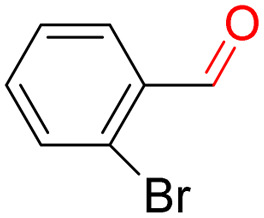	99.9	>99.9	>99.9
6	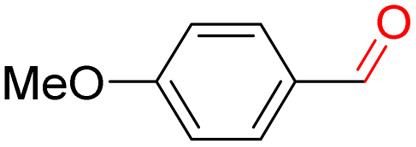	99.9	>99.9	>99.9
7	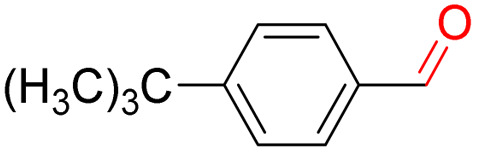	99.9	>99.9	>99.9
8	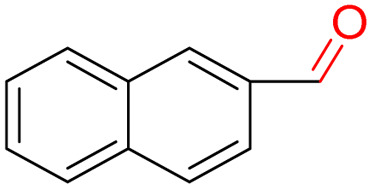	99.9	>99.9	>99.9
9	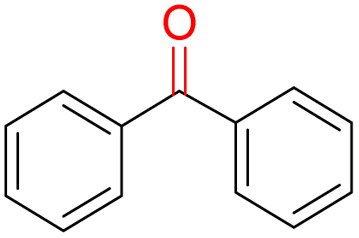	99.9	>99.9	>99.9
10^b^	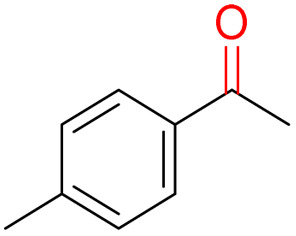	99.9	>99.9	30.5
11^b^	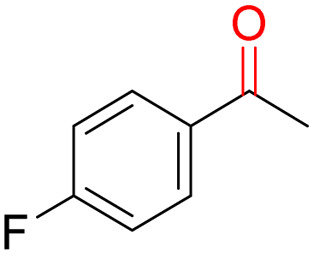	99.9	>99.9	47.3
12	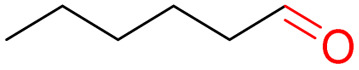	n.d.	—	n.d

a Reaction condition: 5.0 mL of toluene, 0.5 mmol of alcohols, 150.0 mg of N_55_-MnO_2_, 1200 rpm, 25.0 ± 1.0 °C, O_2_ flow 16.0 mL min^−1^ 1.0 bar, reaction time 2.5 h, n. d. not detect.

b Reaction time 6.0 h.

## Conclusions

In conclusion, we have successfully developed a range of N_*y*_-MnO_2_ nanocatalysts capable of directly generating ^1^O_2_ at room temperature for the aerobic oxidation of alcohols. The *in situ* insertion of interstitial N into MnO_2_ within encapsulated aqueous microreactors can lead to O_s_ concentrations of up to 51.1% for N_55_-MnO_2_ and enhance chemisorption of reactants. Consequently, these well-synthesized N_*y*_-MnO_2_ nanocatalysts have demonstrated enhanced redox performance due to their higher surface O_v_ exposure and N-doping. DFT calculations have revealed that the interstitial N and increased O_v_ content facilitates the chemisorption of O_2_, resulting in the generation of sufficient ^1^O_2_. Under aerobic oxidation conditions, the N_55_-MnO_2_ catalyst has achieved alcohol conversion rates exceeding 99.9% and aldehyde selectivity of over 99.9%, without the use of additives. Additionally, the N_55_-MnO_2_ catalyst has displayed a TOF value of 0.14 mol mol_cat_^−1^ h^−1^, 6.7 times higher than that of the N-undoped ε-MnO_2_ catalyst. Furthermore, N_55_-MnO_2_ has shown excellent catalytic stability after ten cycles, remaining robust even after recycling and calcination under an air atmosphere. This study introduces a strategy for fabricating efficient ROS triggers within compartmentalized microdroplets, resulting in the generation of favorable ^1^O_2_ to facilitate the aerobic oxidation of alcohols.

## Experimental

### Synthesis of N_*y*_-MnO_2_ catalyst

A homogeneous oil phase was achieved through vigorous mechanical stirring of sodium dodecyl benzene sulfonate (10.5 g) and xylene (90 mL). Subsequently, a mixture containing manganese(ii) nitrate tetrahydrate (10 mmol), water (5.4 mL), and urea (1, 25, 40, 55, 70 mmol) was gently introduced into the aforementioned oil phase, resulting in the formation of a water-in-oil (W/O) microemulsion. After stirring for 30 min, the resulting microemulsion was transferred to a 100 mL Teflon autoclave and hydrothermally treated at 160 °C for 8 h. Finally, the received gray precursor was dried overnight under vacuum, and heated at 350 °C in a muffle furnace to obtain N_*y*_-MnO_2_ catalyst, where *y* represents the molar amount of urea used.

### Catalytic performance of aerobic oxidation

N_*y*_-MnO_2_ (150 mg), toluene (5 mL), and benzyl alcohol (0.5 mmol) were charged into a Schleck flask, and the temperature was maintained at 25.0 ± 1.0 °C. The reaction mixture was stirred magnetically at 1200 rpm, while O_2_ was continuously bubbled at a rate of 16 mL min^−1^. After several hours of reaction, the mixture was analyzed by gas chromatography (PANNA A91Plus). The used N_*y*_-MnO_2_ was recycled by high-speed centrifugation and subsequently calcined at 350 °C for 4 h.

### ROS trapping experiment

The ROS control experiments followed procedures similar to the aerobic oxidation process. In these experiments, benzyl alcohol and FA were employed as the model substrate and ^1^O_2_ scavenger, respectively. N_*y*_-MnO_2_ (150.0 mg), toluene (5.0 mL), benzyl alcohol (0.5 mmol), and FA (1.0 mmol) were sequentially introduced into a reaction tube, maintaining the temperature at 25.0 ± 1.0 °C. The mixture underwent magnetic stirring (1200 rpm) with a continuous O_2_ flow (16.0 mL min^−1^). Upon completion of the reaction, the conversion of benzyl alcohol was assessed using gas chromatography. Additionally, experiments involving TEMPO, BQ and IPA quenchers were conducted following the aforementioned procedures. The roles of ROS (^1^O_2_, ˙O_2_^−^ and ˙OH) in oxidative reaction were analyzed based on the observed oxidation of benzyl alcohol.

## Author contributions

J. T. and J. C.: conceptualization, methodology, writing – original draft. Y. Z. Q. M. and P. C.: chemical experiments and analysed the data. Y. K., L. F., J. K., and T. H. participated in various aspects of the experiments and discussions. X. H., P. L. and Z. L.: recycling experiments. C. W., Q. K., Y. K., and Y. Y. review & editing, supervision.

## Conflicts of interest

There are no conflicts to declare.

## Supplementary Material

SC-014-D3SC04418A-s001

## References

[cit1] Lu C., Zhang C., Wang P., Zhao Y., Yang Y., Wang Y., Yuan H., Qu S., Zhang X., Song G., Pu K. (2020). Chem.

[cit2] Bloyet C., Sciortino F., Matsushita Y., Karr P. A., Liyanage A., Jevasuwan W., Fukata N., Maji S., Hynek J., D'Souza F., Shrestha L. K., Ariga K., Yamazaki T., Shirahata N., Hill J. P., Payne D. T. (2022). J. Am. Chem. Soc..

[cit3] Bao X., Li H., Wang Z., Tong F., Liu M., Zheng Z., Wang P., Cheng H., Liu Y., Dai Y., Fan Y., Li Z., Huang B. (2021). Appl. Catal., B.

[cit4] Liu X., Yu H., Huang J., Su J., Xue C., Zhou X., He Y., He Q., Xu D., Xiong C., Ji H. (2022). Chem. Sci..

[cit5] Kalaitzakis D., Bosveli A., Sfakianaki K., Montagnon T., Vassilikogiannakis G. (2021). Angew. Chem., Int. Ed..

[cit6] Wu W., Han C., Zhang Q., Zhang Q., Li Z., Gosztola D. J., Wiederrecht G. P., Wu M. (2018). J. Catal..

[cit7] Ke Q., Fang S., Tang J., Li F., Ning C., Tang Z., Ling Q., Liu X., Cui P. (2022). ChemPhotoChem.

[cit8] Kong L., Fang G., Xi X., Wen Y., Chen Y., Xie M., Zhu F., Zhou D., Zhan J. (2021). Chem. Eng. J..

[cit9] Ma H., Long S., Cao J., Xu F., Zhou P., Zeng G., Zhou X., Shi C., Sun W., Du J., Han K., Fan J., Peng X. (2021). Chem. Sci..

[cit10] Lin C., Bachilo S. M., Weisman R. B. (2020). J. Am. Chem. Soc..

[cit11] Gao R., Mei X., Yan D., Liang R., Wei M. (2018). Nat. Commun..

[cit12] Singh N., Kumar P., Kumar R., Riaz U. (2019). Ind. Eng. Chem. Res..

[cit13] Chen Y., Wang Z., Wang H., Lu J., Yu S., Jiang H. (2017). J. Am. Chem. Soc..

[cit14] Long R., Mao K., Ye X., Yan W., Huang Y., Wang J., Fu Y., Wang X., Wu X., Xie Y., Xiong Y. (2013). J. Am. Chem. Soc..

[cit15] Huang J., He S., Goodsell J. L., Mulcahy J. R., Guo W., Angerhofer A., Wei W. D. (2020). J. Am. Chem. Soc..

[cit16] Zhang W., Huang W., Jin J., Gan Y., Zhang S. (2021). Appl. Catal., B.

[cit17] Tang J., Cao Y., Ruan F., Li F., Jin Y., Ha M. N., Han X., Ke Q. (2020). Ind. Eng. Chem. Res..

[cit18] Chen J., Tang H., Huang M., Yan Y., Zhang J., Liu H., Zhang J., Wang G., Wang R. (2021). ACS Appl. Mater. Interfaces.

[cit19] Ruan F., Li F., Dong Z., Ke Q., Jin Y., Zhan W., Ha M. N., Tang J. (2021). Green Synth. Catal..

[cit20] Koutani M., Hayashi E., Kamata K., Hara M. (2022). J. Am. Chem. Soc..

[cit21] Ke Q., Jin Y., Ruan F., Ha M. N., Li D., Cui P., Cao Y., Wang H., Wang T., Nguyen V. N., Han X., Wang X., Cui P. (2019). Green Chem..

[cit22] Pérez-Badell Y., Solans-Monfort X., Sodupe M., Montero L. A. (2010). Phys. Chem. Chem. Phys..

[cit23] Oviedo J., Gillan M. J. (2001). Surf. Sci..

[cit24] Hao Y., Liu B., Tian L., Li F., Ren J., Liu S., Liu Y., Zhao J., Wang X. (2017). ACS Appl. Mater. Interfaces.

[cit25] Wang J., Xu X., Liu Y., Wang Z., Wang P., Zheng Z., Cheng H., Dai Y., Huang B. (2020). ChemSusChem.

[cit26] Tan S., Ji Y., Zhao Y., Zhao A., Wang B., Yang J., Hou J. G. (2011). J. Am. Chem. Soc..

[cit27] Setvín M., Aschauer U., Scheiber P., Li Y., Hou W., Schmid M., Selloni A., Diebold U. (2013). Science.

[cit28] Li M., You S., Duan X., Liu Y. (2022). Appl. Catal., B.

[cit29] Kang X., Dong G., Dong T. (2023). ACS Appl. Energy Mater..

[cit30] Zhuo Y., Guo X., Cai W., Shao T., Xia D., Li C., Liu S. (2023). Appl. Catal., B.

[cit31] Yang M., Wu K., Sun S., Duan J., Liu X., Cui J., Liang S., Ren Y. (2023). ACS Catal..

[cit32] Tang J., Zhang Q., Hu K., Zhang P., Wang J. (2017). J. Catal..

[cit33] Liu M., Wang Q., Liu Z., Zhao Y., Lai X., Bi J., Gao D. (2020). Chem. Eng. J..

[cit34] Vardhan P. V., Idris M. B., Liu H. Y., Sivakkumar S. R., Balaya P., Devaraj S. (2018). J. Electrochem. Soc..

[cit35] He T., Zeng X., Rong S. (2020). J. Mater. Chem. A.

[cit36] Frankcombe T. J., Liu Y. (2023). Chem. Mater..

[cit37] Qi G., Liu X., Li C., Wang C., Yuan Z. (2019). Angew. Chem., Int. Ed..

[cit38] Liu L., Liu R., Xu T., Zhang Q., Tan Y., Zhang Q., Ding J., Tang Y. (2020). Inorg. Chem..

[cit39] Rong S., Li K., Zhang P., Liu F., Zhang J. (2018). Catal. Sci. Technol..

[cit40] Huang Y., Liu Y., Wang W., Chen M., Li H., Lee S., Ho W., Huang T., Cao J. (2020). Appl. Catal., B.

[cit41] Morales F., Grandjean D., Mens A., Groot F. M. F., Weckhuysen B. M. (2006). J. Phys. Chem. B.

[cit42] Xu Y., Dhainaut J., Rochard G., Dacquin J., Mamede A., Giraudon J., Lamonier J., Zhang H., Royer S. (2020). Chem. Eng. J..

[cit43] Tang J., Jiao B., Chen W., Ruan F., Li F., Cui P., Wan C., Ha M. N., Nguyen V. N., Ke Q. (2022). Nano Res..

[cit44] Jin Y., Li F., Cui P., Yang Y., Ke Q., Ha M. N., Zhan W., Ruan F., Wan C., Lei Z., Nguyen V. N., Chen W., Tang J. (2021). Nano Res..

[cit45] Ke Q., Yi D., Jin Y., Lu F., Zhou B., Zhan F., Yang Y., Gao D., Yan P., Wan C., Cui P., Golberg D., Yao J., Wang X. (2020). ACS Sustain. Chem. Eng..

[cit46] Li H., Zhang Y., Tang J., Huang G., Cui P., Ke Q. (2022). Green Synth. Catal..

[cit47] Yu J., Zeng T., Wang H., Zhang H., Sun Y., Chen L., Song S., Li L., Shi H. (2020). Chem. Eng. J..

[cit48] Li H., Shang J., Ai Z., Zhang L. (2015). J. Am. Chem. Soc..

[cit49] Lin Y., Liu Z., Yu L., Zhang G., Tan H., Wu K., Song F., Mechler A. K., Schleker P. P. M., Lu Q., Zhang B., Heumann S. (2021). Angew. Chem., Int. Ed..

[cit50] Li F., Tang J., Ke Q., Guo Y., Ha M. N., Wan C., Lei Z., Gu J., Ling Q., Nguyen V. N., Zhan W. (2021). ACS Catal..

[cit51] Li J., Wang X., Tian J., Zhang X., Shi F. (2023). Rare Met..

[cit52] He B., Jin H., Wang Y., Fan C., Wang Y., Zhang X., Liu J., Li R., Liu J. (2022). Rare Met..

[cit53] Siemer N., Lüken A., Zalibera M., Frenzel J., Muñoz-Santiburcio D., Savitsky A., Lubitz W., Muhler M., Marx D., Strunk J. (2018). J. Am. Chem. Soc..

[cit54] Xia T., Gong W., Chen Y., Duan M., Ma J., Cui X., Dai Y., Gao C., Xiong Y. (2022). Angew. Chem., Int. Ed..

