# Supplementary material for: Spontaneous generation of singlet oxygen on microemulsion-derived manganese oxides with rich oxygen vacancies for efficient aerobic oxidation
Source: Chem Sci. 2023 Oct 12;14(46):13402–9. doi: 10.1039/d3sc04418a (PMC10685315; doi:10.1039/d3sc04418a)
Supplement: SC-014-D3SC04418A-s001 [file SC-014-D3SC04418A-s001.pdf]

This journal is © The Royal Society of Chemistry 2023

1. Experimental procedures

1.1 Materials

Sodium dodecyl benzene sulfonate (SDBS, AR grade), ethanol (≥99.5%), manganese (II) nitrate tetrahydrate ($Mn(NO_3)_2{\cdot}4H_2O$, 98%), sodium bicarbonate ($NaHCO_3$, ≥99.8%), urea (99%), potassium permanganate ($KMnO_4$, 99.0%), commercial manganese dioxide (C-MnO_2, ≥90%), and manganese sulfate monohydrate ($MnSO_4{\cdot}H_2O$, 99.0%) were purchased from Aladdin Co. Ltd. Xylene (≥99.0%) and toluene (≥99.5%) were acquired from Sinopharm Chemical Reagent Co., Ltd. 2,2,6,6-Tetramethyl-1-piperidinyloxy (TEMPO, 98%), 1,4-benzoquinone (BQ, ≥98.0%), furfuryl alcohol (FA, 98%), and isopropanol (IPA, ≥99.5%) were obtained from Sigma-Aldrich. 2,2,6,6-Tetramethylpiperidine (TEMP, ≥99%) and 5,5-dimethyl-1-pyrroline *N*-oxide (DMPO, ≥98.0%) used in the experiments were acquired from Dojindo Laboratories. All alcohols used as substrates were sourced from Energy Chemical.

1.2 Characterization

X-ray diffraction (XRD) spectra were obtained using a Bruker D8 Advance powder X-ray diffractometer (Bruker, Germany) equipped with Cu Kα radiation. The scanning angle range was 10-80°, and the scanning rate was 5 °/min. X-ray photoelectron spectroscopy (XPS) spectra were recorded using a Thermo Scientific K-Alpha system (ThermoFischer, USA) with an Al Kα radiation source ($h\nu$=1486.6 eV). Brunauer-Emmett-Teller (BET) surface area data were determined by analyzing adsorption with liquid N_2 at -195.8 ºC using an ASAP 2460 sorption instrument (Micromeritics, USA). Morphology observations of N_y-MnO_2 were conducted using a high-resolution transmission electron microscope (TEM) Talos-F200S (FEI, USA). Hydrogen temperature-programmed reduction with H_2 (H_2-TPR) was carried out using an AMI-300 system (Altamira, USA). Samples were pretreated in N_2 atmosphere and subsequently reduced in 10 vol.% H_2/Ar gas mixture at a flow rate of 30 mL/min. Singlet oxygen ($^1O_2$), superoxide anion ($\cdot O_2^-$), and hydroxyl radical (·OH) generation activated by N_y-MnO_2 were examined using a Bruker A300 electron paramagnetic resonance (EPR) spectroscopy (Bruker, Germany). Fourier Transform infrared (FTIR) spectra were acquired using a Thermo Scientific Nicolet iS50 spectrometer (ThermoFisher, USA). Dynamic light scattering (DLS) analysis was performed using a Malvern Zetasizer Nano ZS90. The conversion of alcohols and the selectivity of target products were determined using Gas chromatography (GC) on a PANNA A91Plus instrument (China).

1.3 Synthesis of ε-MnO_2 by hydrothermal method

The synthesis of micrometer-size ε-MnO_2 without N doping was achieved using a hydrothermal method. A solution was prepared by dissolving $MnSO_4{\cdot}H_2O$ (0.17 g, 1 mmol) in a mixture of deionized water (70 mL) and ethanol (7 mL). Subsequently, $NaHCO_3$ (0.84 g, 10 mmol) was added to the solution with stirring and allowed to stand for 3 h at room temperature. The resulting powder was carefully collected, dried, and then calcined at 350 °C for 4 hours in an air environment to produce ε-MnO_2 catalyst.

1.4 Synthesis of ε-MnO_2 nanocatalysts (ε-MnO_2-n) by microemulsion method

ε-MnO_2 nanocatalysts without N doping were synthesized using the microemulsion method. SDBS (10.5 g) was dissolved in xylene (90 mL) with the assistance of ultrasonication. Aqueous solutions of $KMnO_4$ (0.316 g, 2 mmol) in deionized water (1 mL) and $MnSO_4{\cdot}H_2O$ (1.44 g, 8.5 mmol) in deionized water (4.4 mL) were sequentially added to the oil phase mentioned earlier, followed by stirring for 0.5 hours. The resulting microemulsion was subjected to hydrothermal treatment at 160 °C for 12 hours. After drying at 80 °C for 12 hours, the obtained precursors were calcined at temperatures of 300, 350, 400, and 500 °C for 4 hours in an air environment, yielding the ε-MnO_2-n-y nanocatalyst, where y corresponds to the calcination temperature (300, 350, 400, and 500 °C).

1.5 Procedures of commercial activated MnO_2 (C-MnO_2)

The standard procedure for activating C-MnO_2 is as follows: The C-MnO_2 sample was slowly heated to 350 °C at a rate of 2 °C/min and maintained at this temperature for 4 hours in an air atmosphere.

1.6 Procedures for kinetic experiments

In a Schlenk flask, N_y-MnO_2 (150 mg), toluene (5 mL), and benzyl alcohol (0.5 mmol) were combined. The reaction mixture was stirred using a magnetic stirrer at 1200 rpm while continuously bubbling oxygen (O_2) into the solution (16 mL/min). The reaction was conducted at various temperatures (20, 25, 30, and 35 °C), and samples were withdrawn from the reaction mixture at specific times intervals (10, 20, 30, 40, and 50 min) for analysis using GC.

1.7 Procedures for EPR test

The qualitative and quantitative analysis of the generation of reactive oxygen species (ROS), including $^1O_2$, $O_2{\cdot}^-$, and ·OH, was performed using a Bruker A300 EPR spectrometer. The radiation frequency was set at 9.85 GHz, and a power of 20 mW was applied. The standard test procedures for assessing $^1O_2$, $O_2{\cdot}^-$, and ·OH in the absence of light were as follows.

Qualitative analysis of $^1O_2$, $O_2{\cdot}^-$, and ·OH:

For the $^1O_2$ test, 30 mg of the catalyst was sonicated in 2 mL of deionized water to ensure uniform dispersion, followed by bubbling O_2 for 10 minutes. Subsequently, 30 μL of 2,2,6,6-Tetramethylpiperidine (TEMP) aqueous solution (200 mM) was added to the above dispersion. The concentration of $^1O_2$ in the reaction mixture was determined at the end of the experiment. All $^1O_2$ tests were conducted at room temperature without exposure to light.

5,5-Dimethyl-1-pyrroline *N*-oxide (DMPO) was employed as the capture agent for $\cdot O_2^-$ and ·OH. To perform the tests, 30 mg of the catalyst was dispersed in the appropriate solvent. Subsequently, 30 μL DMPO solution (200 mM) was added to the dispersion. It is worth noting that methanol was used as the solvent used for $O_2{\cdot}^-$ testing, while deionized water was employed for ·OH testing. All $O_2{\cdot}^-$and ·OH tests were conducted at room temperature without exposure to light.

Quantitative analysis of $^1O_2$:

For the $^1O_2$ test, 30 mg of catalyst was sonicated in 2 mL of deionized water to ensure uniform dispersion,

followed by bubbling O_2 for 10 minutes. Subsequently, 30 μL of 2,2,6,6-tetramethylpiperidine (TEMP) aqueous solution (at concentrations of 50/100/200/250 mM) was added to the dispersion. Based on the experimental results, the optimal concentration of TEMP solution was found to be 200 mM. All $^1O_2$ tests were conducted at room temperature without exposure to light.

1.8 Procedures for *in-situ* IR test

N_y-MnO_2 nanocatalysts were compressed into sheets and placed inside a chamber with CaF_2 window. The samples underwent pretreatment at 120 °C for 2 hours under a protective N_2 atmosphere, followed by cooling to room temperature. These treated catalysts served as the background for data acquisition. Subsequently, benzyl alcohol (10 μL) was applied to the catalyst surface, and the adsorption data of the substrate were recorded.

For the chemisorption of O_2 onto the catalysts, the pretreatment procedures remained consistent with those described earlier. After collecting the background data, detailed spectra were obtained within an atmosphere of O_2.

1.9 DFT calculation method

All calculations are conducted within the framework of density functional theory (DFT) using projection enhanced plane wave methods, as implemented in the Vienna ab initio simulation package.[1] For the exchange-correlation potential, we adopted the generalized gradient approximation proposed by Perdew, Burke and Ernzerhof.[2] To account for long-range van der Waals interactions, we employed the DFT-D3 method.[3] The plane wave cut-off energy was set to 420 eV. When solving the Kohn-Sham equation iteratively, an energy criterion of 10^{-5} eV was applied. Brillouin zone integration was performed at the Gamma point using a 3×3×1 k-mesh grid. All structures were relaxed until the residual force on each atom decreased to below 0.03 $eV \cdot Å^{-1}$.

2. Figures and Tables

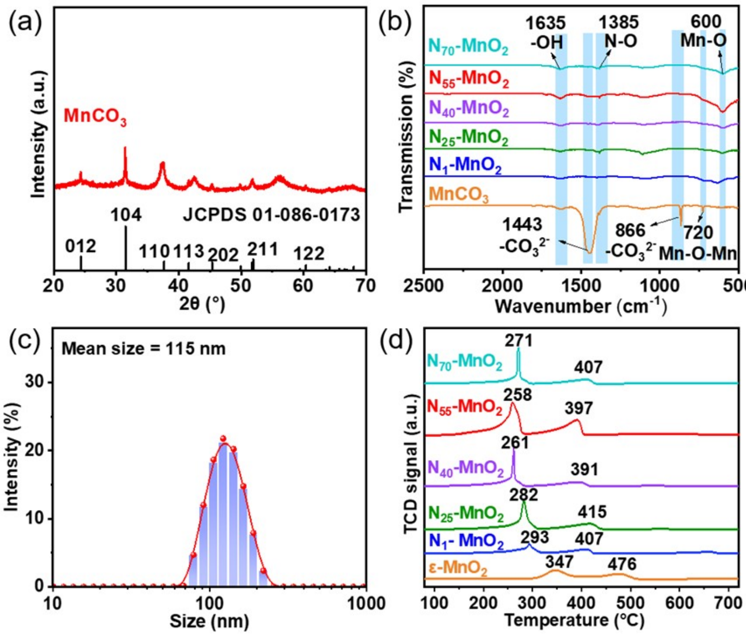

Fig. S1 (a) XRD pattern of prepared catalyst precursor $MnCO_3$; (b) FTIR spectra of N_y-MnO_2 (y=1, 25, 40, 55, 70), and $MnCO_3$; (c) DLS analysis of N_{55}-MnO_2 dispersed in ethanol; (d) H_2-TPR spectra of N_y-MnO_2 (y=1, 25, 40, 55, 70), and ε-MnO_2.

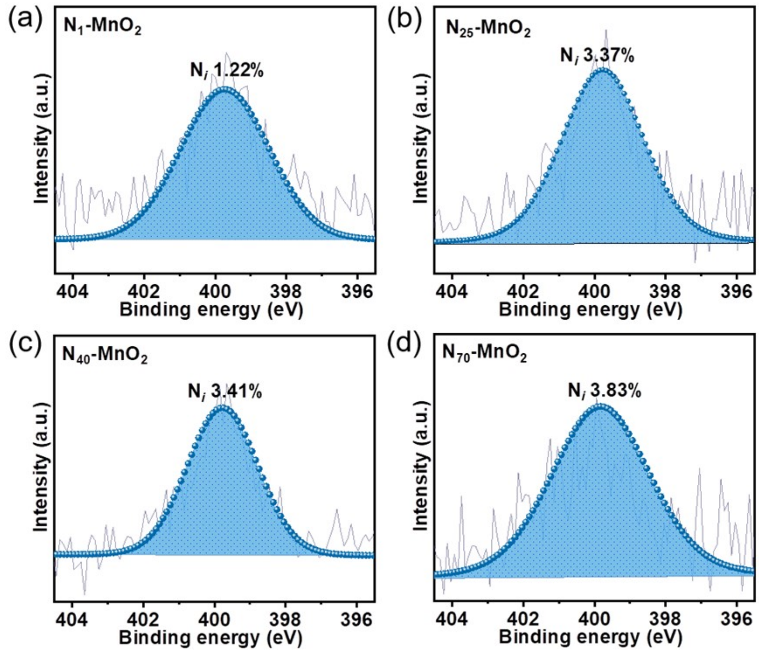

Fig. S2 N 1*s* XPS spectra of (a) N_1-MnO_2, (b) N_{25}-MnO_2, (c) N_{40}-MnO_2, and (d) N_{70}-MnO_2.

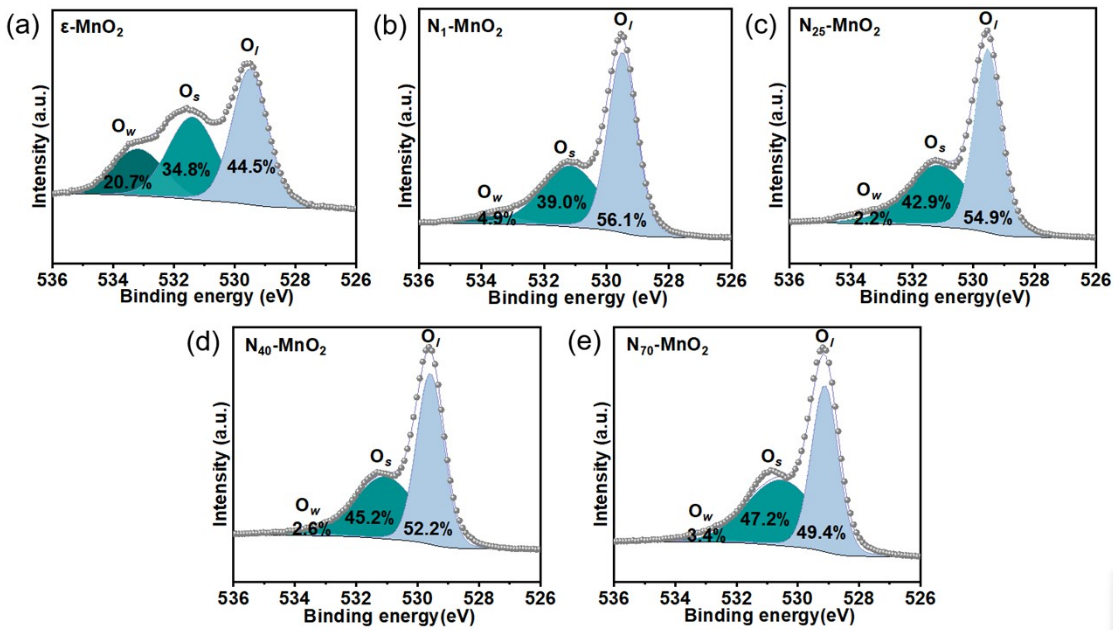

Fig. S3 O 1*s* XPS spectra of (a) ε-MnO_2, (b) N_1-MnO_2, (c) N_{25}-MnO_2, (d) N_{40}-MnO_2, (e) N_{70}-MnO_2. The O1*s* can be fitted using lattice oxygen (O_l), surface oxygen atoms (O_s) in the vicinity of O_v, and oxygen from water adsorption (O_w), corresponding to the binding energies of 529.5, 531.4, and 533.2 eV, respectively.

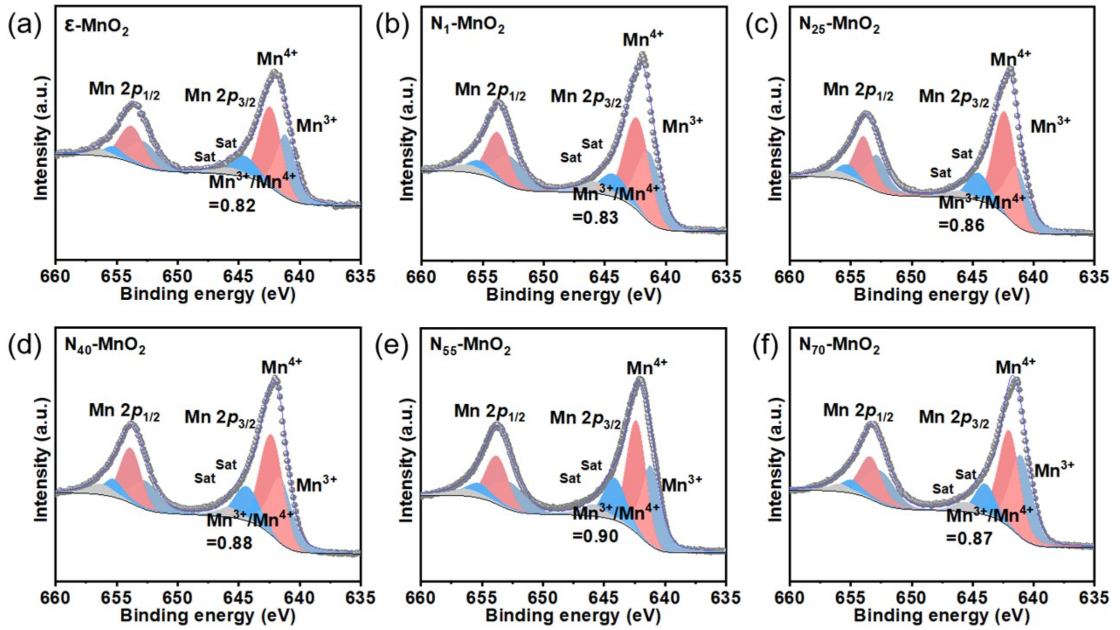

Fig. S4 Mn 2*p* XPS spectra of (a) ε-MnO_2, (b) N_1-MnO_2, (c) N_{25}-MnO_2, (d) N_{40}-MnO_2, (e) N_{55}-MnO_2, and (f) N_{70}-MnO_2.

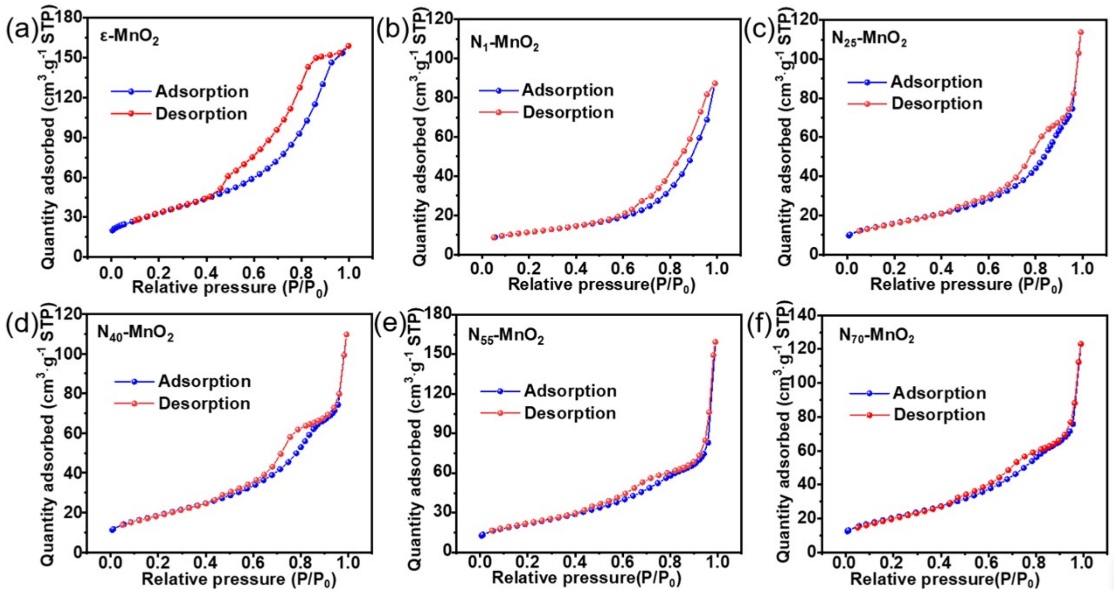

Fig. S5 N_2 adsorption-desorption isotherms of (a) ε-MnO_2, (b) N_1-MnO_2, (c) N_{25}-MnO_2, (d) N_{40}-MnO_2, (e) N_{55}-MnO_2, and (f) N_{70}-MnO_2.

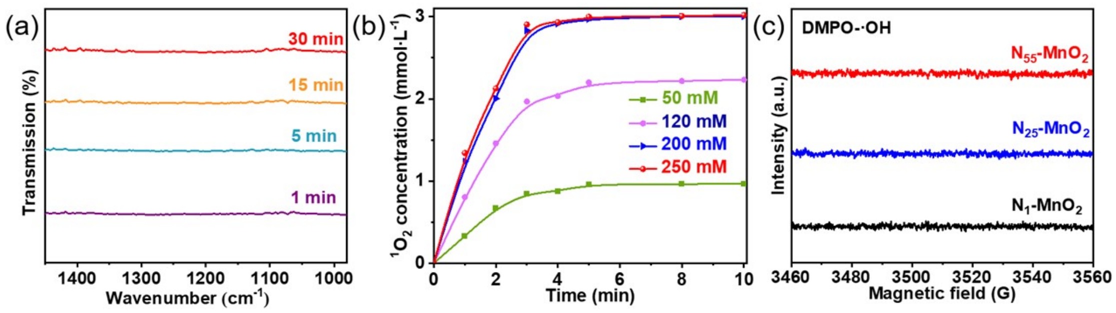

Fig. S6 (a) *In-situ* IR spectra of O_2 adsorbed onto the surface of N_{55}-MnO_2 in O_2 + H_2O vapor atmosphere; (b) $^1O_2$ concentration detected with different TEMP concentrations; (c) EPR spectra of DMPO-·OH triggered by N_1-MnO_2, N_{25}-MnO_2 and N_{55}-MnO_2.

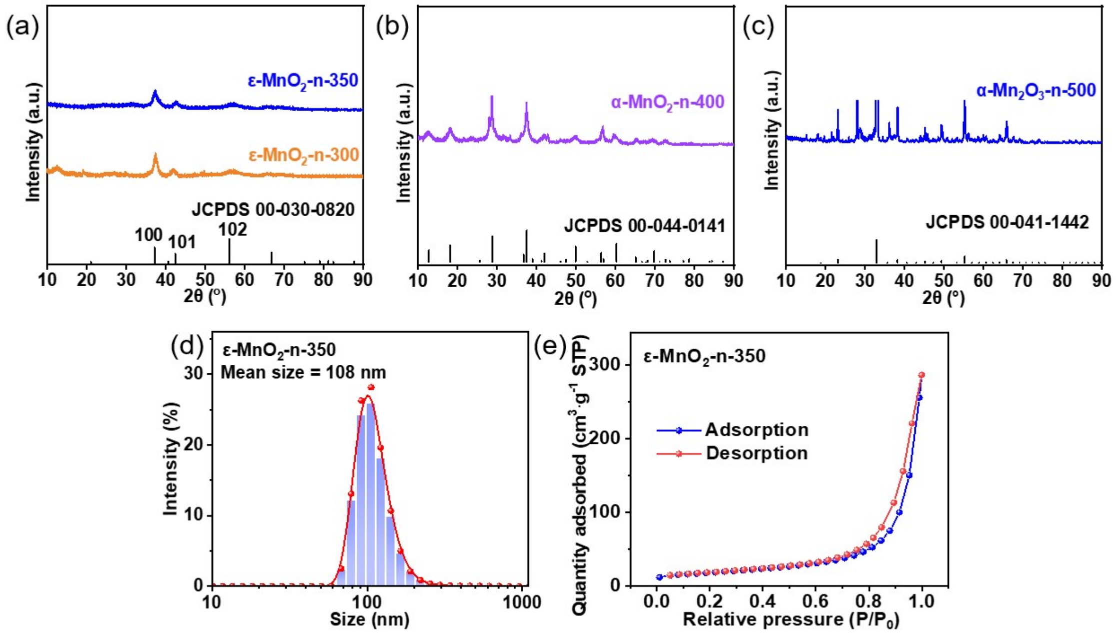

Fig. S7 XRD patterns of (a) ε-MnO_2-n-300 and ε-MnO_2-n-350, (b) α-MnO_2-n-400 and (c) α-Mn_2O_3-n-500; (d) DLS result of ε-MnO_2-n-350; (e) BET result of ε-MnO_2-n-350.

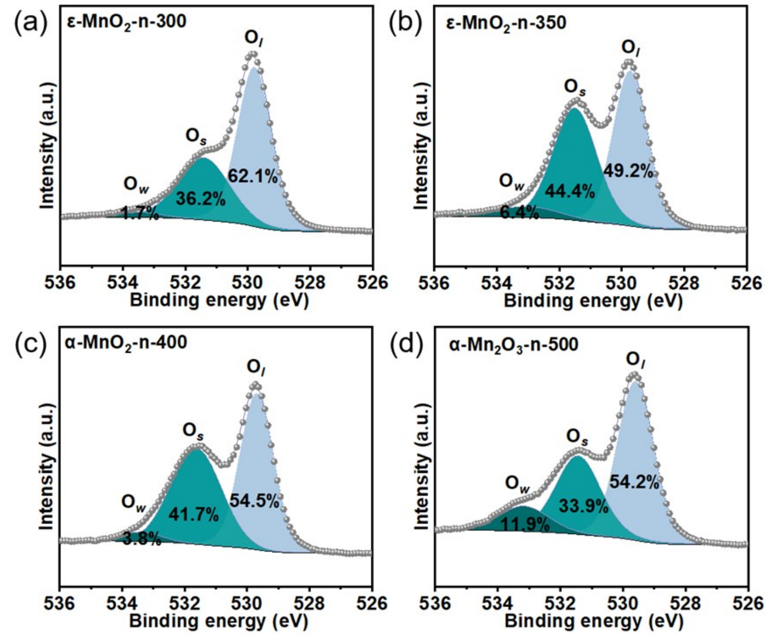

Fig. S8 O1*s* XPS spectra of (a) ε-MnO_2-n-300, (b) ε-MnO_2-n-350, (c) ε-MnO_2-n-400, and (d) α-Mn_2O_3-n-500.

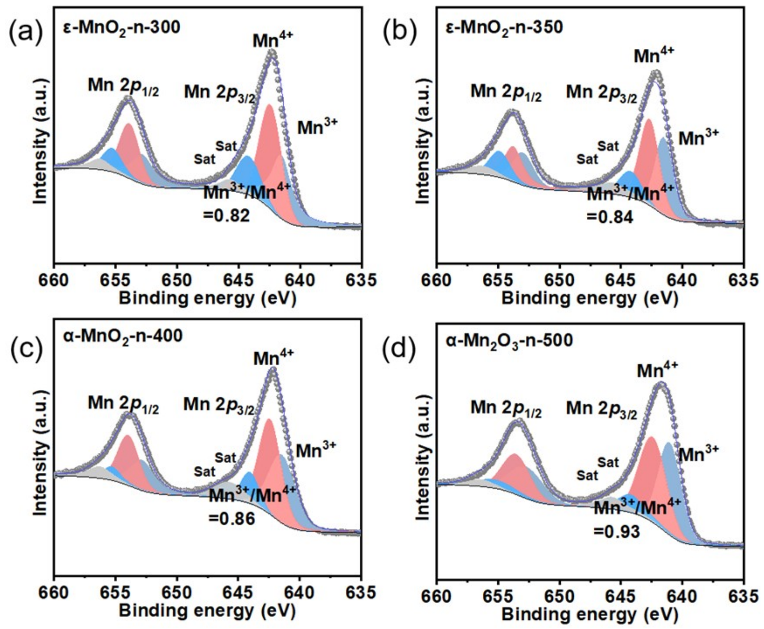

Fig. S9 Mn 2*p* XPS spectra of (a) ε-MnO_2-n-300, (b) ε-MnO_2-n-350, (c) ε-MnO_2-n-400, and (d) α-Mn_2O_3-n-500.

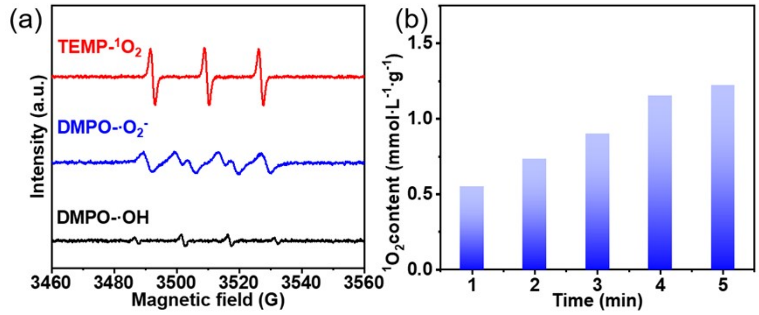

Fig. S10 (a) The EPR characteristic peaks of TEMP-$^1O_2$, DMPO-$\cdot O_2^-$ and DMPO-$\cdot OH$ generated by ε-MnO_2-n-350; (b) $^1O_2$ content in ε-MnO_2-n-350 suspension (TEMP concentration: 200 mM).

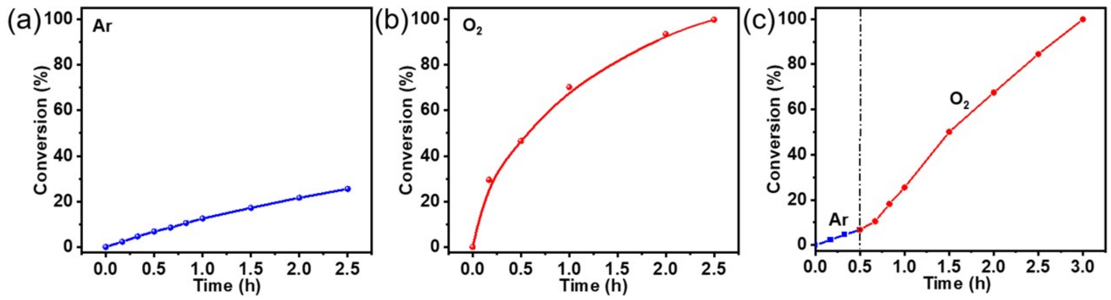

Fig. S11 The comparison of catalytic performance of N_{55}-MnO_2 under (a) Ar, (b) O_2, and (c) Ar + O_2 atmosphere.

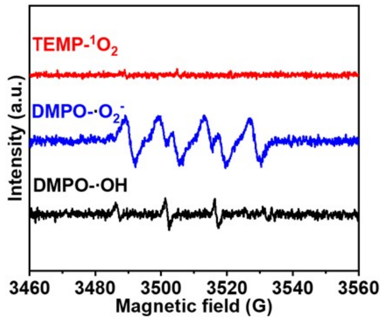

Fig. S12 The EPR characteristic peaks of TEMP-$^1O_2$, DMPO-$\cdot O_2^-$ and DMPO-$\cdot$OH generated by ε-MnO_2.

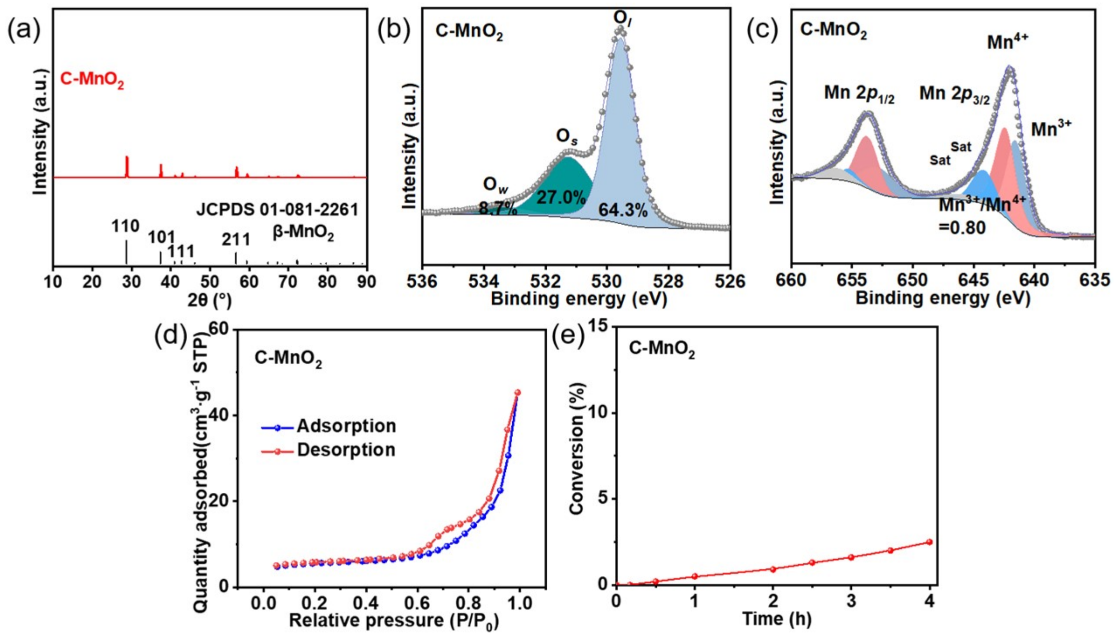

Fig. S13 (a) XRD pattern of C-MnO_2; (b) O 1*s* and (c) Mn 2*p* XPS spectra of C-MnO_2; (d) BET result of C-MnO_2; (e) catalytic performance of activated C-MnO_2 mediated aerobic oxidation of benzyl alcohol.

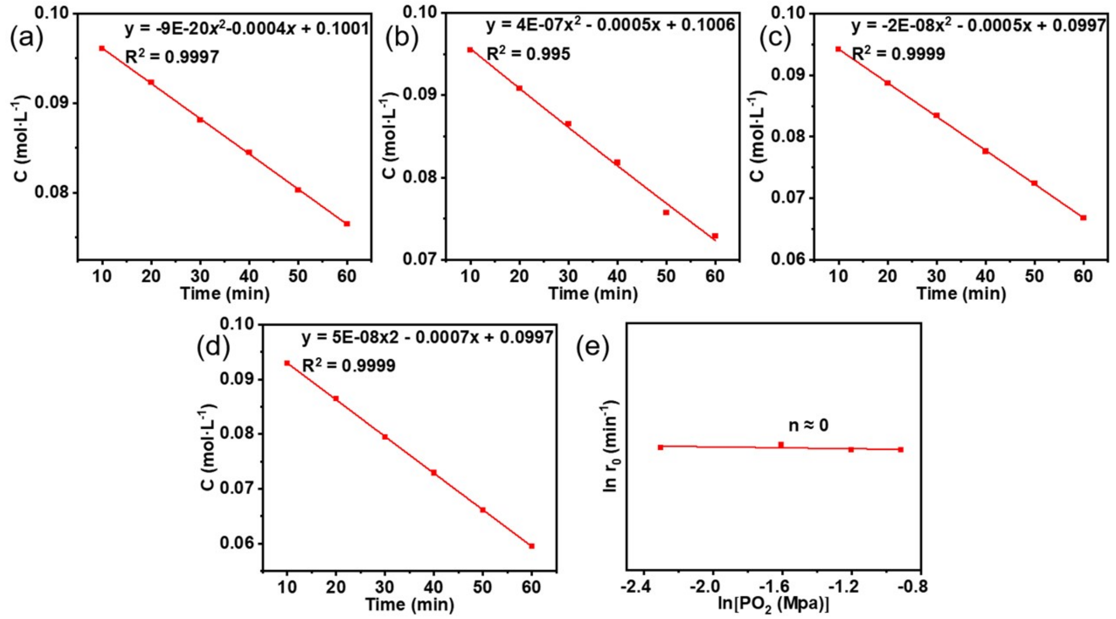

Fig. S14 Polynomial fitting of benzyl alcohol concentration (C) and reaction time (t) under pressure of (a) 0.1 MPa, (b) 0.2 MPa, (c) 0.3 MPa, and (d) 0.4 MPa; (e) relationship between r_0 and O_2 pressure using N_{55}-MnO_2 as a catalyst.

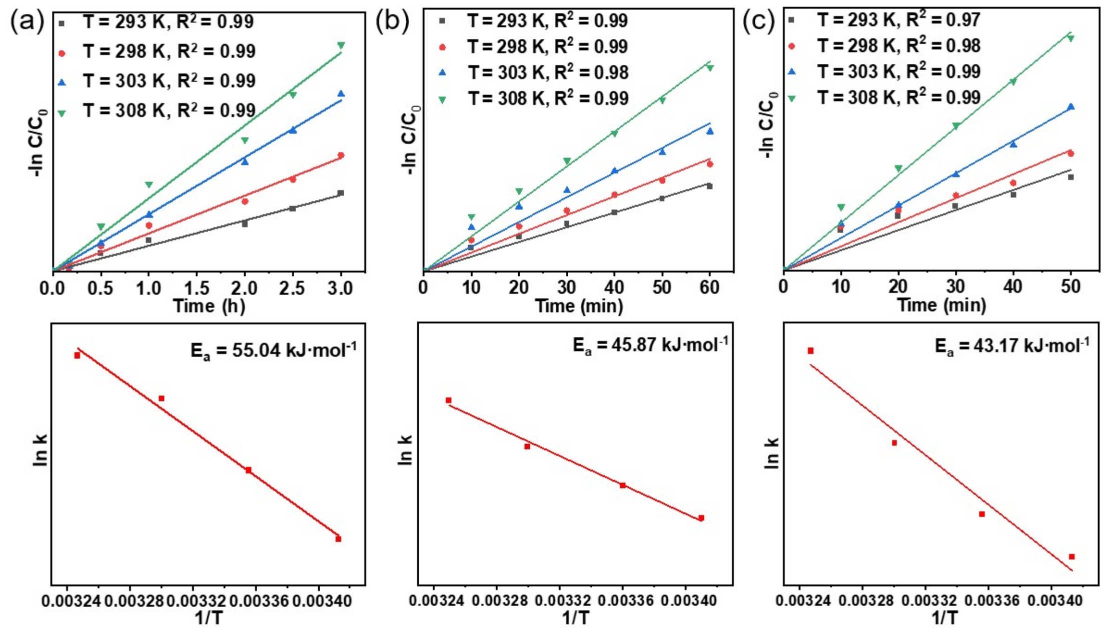

Fig. S15 The kinetic experiements results of (a) N_1-MnO_2, (b) N_{25}-MnO_2, and (c) N_{55}-MnO_2 at different reaction temperature.

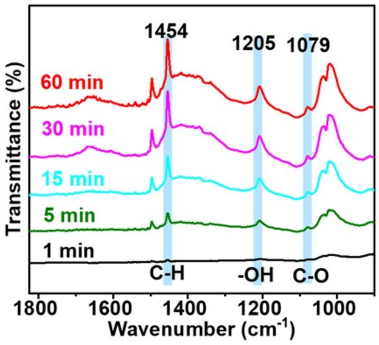

Fig. S16 *In-situ* IR spectra of benzyl alcohol adsorbed on the surface of activated C-MnO_2.

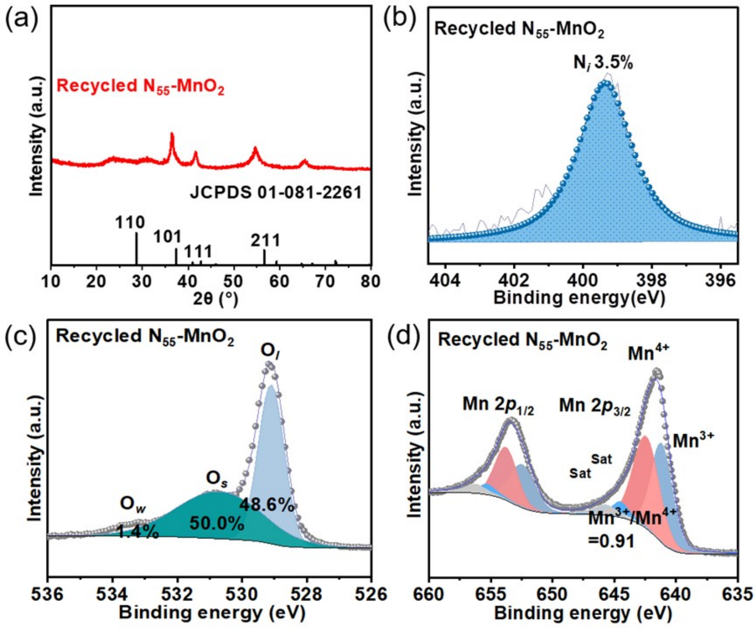

Fig. S17 (a) XRD pattern of recycled N_{55}-MnO_2; (b) N 1*s*, (c) O 1*s*, and (d) Mn 2*p* XPS spectra of recycled N_{55}-MnO_2.

Table S1 XPS analysis results of N_y-MnO_2 (y=1, 25, 40, 55, 70).

Entry	Catalyst	N (mol%)	Mn^{3+}/Mn^{4+}	O_s (%) [a]
1	N_1-MnO_2	1.22	0.83	39.0
2	N_{25}-MnO_2	3.37	0.86	42.9
3	N_{40}-MnO_2	3.41	0.88	45.2
4	N_{55}-MnO_2	3.52	0.90	51.1
5	N_{70}-MnO_2	3.83	0.87	47.2
6	Recycled N_{55}-MnO_2	3.51	0.91	50.0
7	C-MnO_2	/	0.80	27.0
8	ε-MnO_2	/	0.82	34.8
9	ε-MnO_2-n-350	/	0.84	44.4

Table S2 The concentration of O_s and proportion of Mn with different valence of Mn-based catalyst.

Entry	Catalyst	O_s (%)	Mn^{3+}/Mn^{4+}	Ref.
1	Mg-MnO_2	40	0.69	4
2	ε-MnO_2	23	1.27	5
3	ε-MnO_2	21.3	0.45	6
4	N-$Mn_xCo_{3-x}O_4$	45	1.01	7
5	NEG	38.6	/	8
6	MnO_2@GdO_x	45	0.91	9
7	N-MnO_2	37.1	0.83	10
8	8K/MnO_2	36.3	0.56	11
9	MnO_2/AC-N_2	41.5	1.25	12
10	La-MnO_2	34.6	0.35	13
11	N_{55}-MnO_2	51.1	0.90	This work

Table S3 BET results of N_y-MnO_2 (y=1, 25, 40, 55, 70), C-MnO_2, ε-MnO_2, and ε-MnO_2-n-350.

Entry	Catalysts	Surface Area (m^2/g)	Pore Volume (cm^3/g)	Pore Size (nm)
1	N_1-MnO_2	39.7	0.11	10.06
2	N_{25}-MnO_2	57.5	0.18	11.25
3	N_{40}-MnO_2	67.4	0.17	9.19
4	N_{55}-MnO_2	78.7	0.25	11.41
5	N_{70}-MnO_2	74.2	0.19	9.29
6	ε-MnO_2	121.1	0.24	8.1
7	ε-MnO_2-n-350	64.8	0.44	21.35
8	C-MnO_2	17.7	0.07	15.68

Table S4 The control experiment for the N_{55}-MnO_2 catalyzed aerobic oxidation of benzyl alcohol with different quenchers and under various atmospheres.

Entry	Catalysts	Quenchers	Atmosphere	Conversion (%)	Selectivity (%)
1 [a]	N_{55}-MnO_2	/	O_2	>99.9	>99.9
2 [a]	N_{55}-MnO_2	TEMPO	O_2	26	>99.9
3 [a]	N_{55}-MnO_2	BQ	O_2	85	>99.9
4 [a]	N_{55}-MnO_2	FA	O_2	7	>99.9
5 [a]	N_{55}-MnO_2	IPA	O_2	96	>99.9
6 [b]	N_{55}-MnO_2	/	N_2	31	>99.9
7 [c]	N_{55}-MnO_2	/	O_2	>99.9	>99.9
8 [d]	N_{55}-MnO_2	/	O_2	>99.9	>99.9
9 [e]	N_{55}-MnO_2	/	Ar	25.5	>99.9

[a] Reaction conditions: 5.0 mL of toluene, 0.5 mmol of alcohols, 150.0 mg of N_{55}-MnO_2, 1200 rpm, 25.0 ± 1.0 °C, O_2 flow 16.0 mL·min^{-1} 1.0 bar, reaction time 2.5 h, quencher 1.0 mmol;

[b] N_2 bubbling 16.0 mL·min^{-1}, 1.0 bar;

[c] in dark, O_2 flow 16.0 mL·min^{-1} 1.0 bar;

[d] light irradiation, O_2 flow 16.0 mL·min^{-1} 1.0 bar;

[e] Ar bubbling 16.0 mL·min^{-1}, 1.0 bar.

Table S5 Turnover frequency (TOF) values of C-MnO_2, ε-MnO_2, ε-MnO_2-n-350, and N_y-MnO_2 (y=1, 25, 40, 55, 70).

Entry	Catalysts	TOF ($mmol\cdot g^{-1}_{cat}\cdot h^{-1}$)	Selectivity (%)
1	C-MnO_2	0.002	95.0
2	ε-MnO_2	0.019	95.0
3	ε-MnO_2-n-350	0.021	99.0
4	N_1-MnO_2	0.023	99.0
5	N_{25}-MnO_2	0.05	99.2
6	N_{40}-MnO_2	0.10	99.5
7	N_{55}-MnO_2	0.14	>99.9
8	N_{70}-MnO_2	0.12	99.5

TOF: moles of benzyl alcohol converted per mole of catalyst/reaction time (h), calculated at reaction time of 2.0 h.

Table S6 The catalytic conversion of benzyl alcohol using N_{55}-MnO_2 under varying O_2 pressure.

P(MPa) / t (min)	0.1	0.2	0.3	0.4
10	3.9	4.5	5.8	7.1
20	7.7	9.2	11.3	13.6
30	11.9	13.5	16.6	20.6
40	15.5	18.2	22.4	27.1
50	19.7	24.3	27.6	33.9
60	23.5	27.1	33.2	40.5

Reaction temperature: 25.0 ± 1.0 °C

Table S7 The correlation between the benzyl alcohol concentration in the reaction mixture and the O_2 pressure (P).

P(MPa) / t (min)	0.1	0.2	0.3	0.4
10	0.0961	0.0955	0.0942	0.0929
20	0.0923	0.0908	0.0887	0.0864
30	0.0881	0.0865	0.0834	0.0794
40	0.0845	0.0818	0.0776	0.0729
50	0.0803	0.0757	0.0724	0.0661
60	0.0765	0.0729	0.0668	0.0595

Reaction temperature: 25.0 ± 1.0 °C

Table S8 The initial reaction rate (r_0) for the oxidation of benzyl alcohol catalyzed by N_{55}-MnO_2 at varying O_2 pressure.

Entry	P (MPa)	r_0
1	0.1	0.1001
2	0.2	0.1000
3	0.3	0.0997
4	0.4	0.0997

Experiment analysis: We conducted the oxidation of benzyl alcohol was conducted at different O_2 pressures (P, ranging from 0.1-0.4 MPa). The detailed data regarding the conversion of benzyl alcohol are provided in **Table S6**, while the corresponding benzyl alcohol concentration (C) was shown in **Table S7**. In **Fig. S14**, we performed polynomial fitting on the C versus reaction time (t) to calculate r_0 ($r=-dC/dt$).[14-16] The polynomial derivative in **Figure S12** yields r_0 at $t = 0$, as shown in **Table S8**. To assess the influence of O_2 pressure, we fitted a curve relating O_2 pressure and r_0, resulting in an approximate reaction order (n) of 0 (**Fig. S14e**). These results indicate that the reaction rate is nearly independent of O_2 pressure.

Table S9 The results of N_{55}-MnO_2-catalyzed aerobic oxidation of benzyl alcohol.

Entry	t (min)	T (K)	Oxidant	Conversion (%)	Selectivity (%)
1	10	303	O_2	10.05	>99.9
2	20	303	O_2	13.8	>99.9
3	30	303	O_2	19.65	>99.9
4	40	303	O_2	24.9	>99.9
5	50	303	O_2	31.2	>99.9

Table S10 The kinetic data for the N_{55}-MnO_2-catalyzed aerobic oxidation of benzyl alcohol.

Entry	t (min)	Conversion (%)	C_0 (mol·L^{-1})	C (mol·L^{-1})	$\ln\frac{C_{A,0}}{C_A}$
1	0	0	0.1	0.1	0
2	10	10.05	0.1	0.08995	0.10592
3	20	13.8	0.1	0.0862	0.1485
4	30	19.65	0.1	0.08035	0.21878
5	40	24.9	0.1	0.0751	0.28634
6	50	31.2	0.1	0.0688	0.37397

Experiment analysis: The results of the oxidation of benzyl alcohol by N_{55}-MnO_2 at 303 K are presented in the **Tables S9** and **S10**. We assume that the oxidation reaction follows a first-order kinetics model. If there is a linear relationship between $\ln\frac{C_{A,0}}{C_A}$ and t, then the above assumption is valid.[17,18] A linear regression analysis yielded a correlation coefficient (R^2) of 0.99 and a reaction rate constant of k=0.0074 s^{-1}, confirming that the reaction indeed follows first-order kinetics. The activation energies (E_a) of N_1-MnO_2, N_{25}-MnO_2 and N_{55}-MnO_2 catalysts were calculated using the Arrhenius equation based on kinetic data obtained at different reaction temperatures (**Fig. S15** and **Tables S11-S1**3). The calculated activation energies were 55.04 kJ·mol^{-1}, 45.87 kJ·mol^{-1}, 43.17 kJ·mol^{-1}, respectively.

Table S11 The kinetic data for the N_1-MnO_2-catalyzed aerobic oxidation of benzyl alcohol.

Entry	T (K)	k (min^{-1})	R^2
1	293	0.0392	0.99
2	298	0.0583	0.99
3	303	0.0879	0.99
4	308	0.1125	0.99

Table S12 The kinetic data for the N_{25}-MnO_2-catalyzed aerobic oxidation of benzyl alcohol.

Entry	T (K)	k (min^{-1})	R^2
1	293	0.0025	0.99
2	298	0.0032	0.99
3	303	0.0043	0.98
4	308	0.006	0.99

Table S13 The kinetic data for the N_{55}-MnO_2-catalyzed aerobic oxidation of benzyl alcohol.

Entry	T (K)	k (min^{-1})	R^2
1	293	0.0046	0.97
2	298	0.0055	0.98
3	303	0.0074	0.99
4	308	0.0109	0.99

Table S14 Comparison of TOF values between N_{55}-MnO_2 and the reported catalysts.

Entry	Catalyst	Oxidant	Alcohol	Light	Oxygen species	T (°C)	t (h)	Conv. (%)	Sel. (%)	TOF ($mol\cdot mol_{cat}^{-1}\cdot h^{-1}$)	Ref.
1	Mg-OMS-1(MnO_2)	O_2 (1 bar)	BA	/	/	50	24	92	>99.9	0.08	4
2	ε-MnO_2	O_2 (10 bar), $NaHCO_3$	HMF	/	/	100	24	74	>99.9	0.01	5
3	N-$CoMn_2O_4$	Air	BA	λ>420 nm	$^1O_2$/$\cdot O_2^-$	30	0.5	>99.9	>99.9	1.2	19
4	TiO_2/Ti_3C_2	O_2 (1 bar)	BA	λ>420 nm	$\cdot O_2^-$	15	5	97	98	0.01	20
5	N-MnO_2	O_2 (1 bar)	HMF	/	/	25	6	>99.9	>99.9	0.05	21
6	N-MnO_2	O_2 (1 bar)	BA	/		30	4	>99.9	>99.9	0.07	22
7	ov-Bi_2O_3	O_2 (1 bar)	BA	UV-vis	$^1O_2$	25	5	41	89	0.04	23
8	TTEPY (pyridinium)	Air	PNA	λ=395 nm	/	25	5	90	94	/	24
9	β-MnO_2	O_2 (1 bar)	BA	/	/	50	5	92	>99.9	0.13	25
10	Co_3O_4	O_2 (1 bar)	BA	/	/	100	3	97.5	>99.9	0.10	26
11	N_{55}-MnO_2	O_2 (1 bar)	BA	/	$^1O_2$	25	2	93.6	>99.9	0.14	This work

BA: benzyl alcohol; HMF: 5-Hydroxymethylfurfural; PNA: p-nitrobenzyl alcohol.

Reference:

1. G. Kresse and D. Joubert, *Phys. Rev. B*, 1999, **59**, 1758-1775.
2. J. P. Perdew, K. Burke and M. Ernzerhof, *Phys. Rev. lett.*, 1996, **77**, 3865-3868.
3. S. Grimme, J. Antony, S. Ehrlich and H. Krieg, *J. Chem. Phys.*, 2010, **132**, 154104.
4. M. Koutani, E. Hayashi, K. Kamata and M. Hara, *J. Am. Chem. Soc.*, 2022, **144**, 14090-14100.
5. E. Hayashi, Y. Yamaguchi, K. Kamata, N. Tsunoda, Y. Kumagai, F. Oba and M. Hara, *J. Am. Chem. Soc.*, 2019, **141**, 890-900.
6. R. Yang, Z. Guo, L. Cai, R. Zhu, Y. Fan, Y. Zhang, P. Han, W. Zhang, X. Zhu, Q. Zhao, Z. Zhu, C. Chan and Z. Zeng, *Small*, 2021, **17**, 2103052.
7. Y. Jin, F. Li, P. Cui, Y. Yang, Q. Ke, M. N. Ha, W. Zhan, F. Ruan, C. Wan, Z. Lei, V.N. Nguyen, W. Chen and J. Tang, *Nano Res.*, 2021, **14**, 2637-2643.
8. Z. Zhang, S. Li, B. Zhao, X. Zhang, X. Wang, Z. Wen, S. Ji and J. Sun, *J. Phys. Chem. C*, 2021, **125**, 20195-20203.
9. L. Liu, R. Liu, T. Xu, Q. Zhang, Y. Tan, Q. Zhang, J. Ding and Y. Tang, *Inorg. Chem.*, 2020, **59**, 14407-14414.
10. T. He, X. Zeng, S. Rong, *J. Mater. Chem. A*, 2020, **8**, 8383-8396.
11. S. Rong, K. Li, P. Zhang, F. Liu and J. Zhang, *Catal. Sci. Technol.*, 2018, **8**, 1799-1812.
12. Y. Huang, Y. Liu, W. Wang, M. Chen, H. Li, S. Lee, W. Ho, T. Huang and J. Cao, *Appl. Catal. B-Environ.*, 2020, **278**, 119294.
13. L. Yu, H. Chen, Z. Wen, M. Jin, X. Ma, Y. Li, Y. Sang, M. Chen and Y. Li, *Ind. Eng. Chem. Res.*, 2021, **60**, 1624-1632.
14. V. D. Makwana, Y. C. Son, A. R. Howell, S. L. Suib, *J. Catal.*, 2002, **210**, 46-52.
15. L. Kang, X. Liu, A. Wang, L. Li, Y. Ren, X. Li, X. Pan, Y. Li, X. Zong, H. Liu, A. I. Frenkel, T. Zhang, *Angew. Chem. Int. Ed.*, 2020, **59**, 12909-12916.
16. A. Abad, A. Corma, H. García, *Chem. Eur. J.*, 2008, **14**, 212-222.
17. L. Zhang, R. Chen, Y. Tu, X. Gong, X. Cao, Q. Xu, Y. Li, B. Ye, Y. Ye and J. Zhu, *ACS Catal.*, 2023, **13**, 2202-2213.
18. Q. Wang, L. Chen, S. Guan, X. Zhang, B. Wang, X. Cao, Z. Yu, Y. He, D. G. Evans, J. Feng and D. Li, *ACS Catal.*, 2018, **8**, 3104-3115.
19. H. Li, Y. Zhang, J. Tang, G. Huang, P. Cui and Q. Ke, *Green Syn. Catal.*, 2022, https://doi.org/10.1016/j.gresc.2022.09.005.
20. X. Bao, H. Li, Z. Wang, F. Tong, M. Liu, Z. Zheng, P. Wang, H. Cheng, Y. Liu, Y. Dai, Y. Fan, Z. Li and B. Huang, *Appl. Catal. B-Environ.*, 2021, **286**, 119885.
21. Q. Ke, Y. Jin, F. Ruan, M.N. Ha, D. Li, P. Cui, Y. Cao, H. Wang, T. Wang, X. Han, X. Wang and P. Cui, *Green Chem.*, 2019, **21**, 4313-4318.

22. F. Ruan, F. Li, Z. Dong, Q. Ke, Y. Jin, W. Zhan, M.N. Ha and J. Tang, *Green Syn. Catal.*, 2021, **2**, 38-44.

23. J. Wang, X. Xu, Y. Liu, Z. Wang, P. Wang, Z. Zheng, H. Cheng, Y. Dai and B. Huang, *ChemSusChem*, 2020, **13**, 3488-3494.

24. S. Ma, J. Cui, C. Rao, M. Jia, Y. Chen and J. Zhang, *Green Chem.*, 2021, **23**, 1337-1343.

25. Y. Yamaguchi, R. Aono, E. Hayashi, K. Kamata and M. Hara, *ACS Appl. Mater. Interfaces*, 2020, **12**, 36004-36013.

26. K. Li, Y. Pei, P. Xiao, Z. He, S. A. C. Carabineiro, H. Jiang and J. Zhu, *ACS Appl. Nano Mater.*, 2022, **5**, 3722-3732.
